# Cdk1 phosphorylates the Rac activator Tiam1 to activate centrosomal Pak and promote mitotic spindle formation

**DOI:** 10.1038/ncomms8437

**Published:** 2015-06-16

**Authors:** Helen J. Whalley, Andrew P. Porter, Zoi Diamantopoulou, Gavin R. M. White, Eduardo Castañeda-Saucedo, Angeliki Malliri

**Affiliations:** 1 Cell Signalling Group, Cancer Research UK Manchester Institute, The University of Manchester, Manchester M20 4BX, UK

## Abstract

Centrosome separation is critical for bipolar spindle formation and the accurate segregation of chromosomes during mammalian cell mitosis. Kinesin-5 (Eg5) is a microtubule motor essential for centrosome separation, and Tiam1 and its substrate Rac antagonize Eg5-dependent centrosome separation in early mitosis promoting efficient chromosome congression. Here we identify S1466 of Tiam1 as a novel Cdk1 site whose phosphorylation is required for the mitotic function of Tiam1. We find that this phosphorylation of Tiam1 is required for the activation of group I p21-activated kinases (Paks) on centrosomes in prophase. Further, we show that both Pak1 and Pak2 counteract centrosome separation in a kinase-dependent manner and demonstrate that they act downstream of Tiam1. We also show that depletion of Pak1/2 allows cells to escape monopolar arrest by Eg5 inhibition, highlighting the potential importance of this signalling pathway for the development of Eg5 inhibitors as cancer therapeutics.

## Abstract (web summary)

Centrosome separation, promoted by the kinesin Eg5, is antagonized by the guanine nucleotide exchange factor Tiam1 through an unknown mechanism. Here Whalley *et al*. show that Tiam1 is phosphorylated by cyclin-dependent kinase 1 in prophase, leading to downstream activation of p21-activated kinases (PAKs).

## Graphical abstract

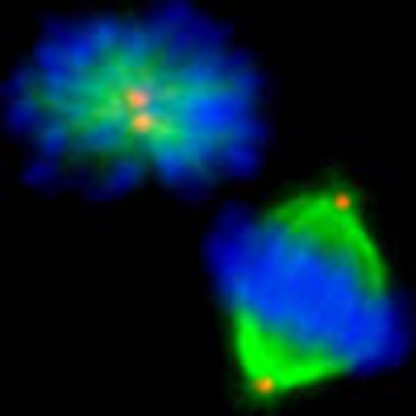

Accurate segregation of chromosomes during mitosis requires formation of a bipolar spindle, which in mammalian cells relies to a large extent on the centrosomes^1^. Following initial Nek2-dependent centrosome disjunction in late G2 (ref. 2), the centrosomes can separate before nuclear envelope breakdown (NEBD) in prophase and post-NEBD in prometaphase. Many mechanisms appear to contribute to centrosome separation after NEBD^3^, but most notable is the plus-end-directed kinesin Eg5, whose microtubule (MT)-sliding activity is essential for centrosome separation in prometaphase across many species^4^ and which also functions in the less-understood prophase pathway in mammalian cells^5^,^6^,^7^. The importance of Eg5 for centrosome separation in both phases is demonstrated by the monopolar spindles and mitotic arrest resulting from its inhibition^8^,^9^, making Eg5 an attractive candidate for anticancer therapy^10^.

Over recent years it has become apparent that forces that oppose centrosome separation are also important to create the correct balance to allow efficient bipolar spindle assembly and chromosome alignment^7^,^11^. Proteins known to produce these forces after NEBD include the minus-end directed kinesins HSET^12^ and dynein^5^, whose inhibition or depletion allows cells to more easily form bipolar spindles under Eg5 inhibition. More recently, we identified the guanine-nucleotide exchange factor (GEF) Tiam1 and its substrate Rac as the first signalling module to counteract Eg5 in prophase^7^. Tiam1 has multiple cellular roles including migration, cell-cell adhesion and survival^13^, and is required for Ras-induced tumorigenesis *in vivo*^14^. We found that Tiam1 also localizes to mitotic centrosomes, and that its depletion leads to increased intercentrosomal distance in early mitosis, a higher incidence of chromosome congression errors and a decreased sensitivity to mitotic arrest by Eg5 inhibitors. Importantly, we showed that the chromosome congression errors were a result of the increased intercentrosomal distance, as they were rescued by re-adjusting the centrosomal distance with low concentrations of Eg5 inhibitor^7^. The mitotic role of Tiam1 may help to explain, at least in part, why the few Ras-induced skin tumours that form in *Tiam1*-knockout mice more frequently undergo malignant progression^14^, as mis-segregation of chromosomes could increase chromosomal instability (CIN), a hallmark of tumourigenesis. Despite the potential importance of this mitotic role, it is not known how Tiam1 operates to antagonize Eg5.

The initiation of mitosis and regulation of centrosome separation is controlled by the mitotic kinases cyclin-dependant kinase 1 (Cdk1), polo-like kinase 1 (Plk1) and Aurora A^15^. In mammals, mitotic entry requires Cdk1 associated with both A and B type cyclins, although cyclin B1 is more directly associated with regulating mitotic entry and exit^16^,^17^. Activation of Cdk1-cyclin B at the G2/M transition occurs on centrosomes^18^ and depends on the activities of Plk1 and Aurora A, which are also present on the centrosomes in late G2^19^. Active Cdk1 stimulates Eg5 both by contributing to its proper localization through phosphorylating Nek9^20^ and by phosphorylating the kinesin directly in its C-terminal tail domain, which stimulates MT binding^21^. Cdk1 is also thought to promote centrosome separation by initiating destablization of interphase astral MTs, which normally inhibit centrosome separation in G2 (ref. 22). Active Cdk1 also primes certain substrates (with the sequence S-S/T-P) for phosphorylation by Plk1 by creating a docking site for its polo box domain (PBD)^23^. One such protein is Nek9, whose sequential phosphorylation by Cdk1 and Plk1 is required for it to activate Nek6/7, which promote Eg5 centrosomal localization by phosphorylating the kinesin on S1033 (ref. 20). The importance of Plk1 in promoting centrosome separation through proper Eg5 localization is well established^20^,^22^,^24^, but Plk1 is also required for the earlier stage of initial centrosome dysjunction, as it phosphorylates the Hippo pathway component Mst2 that in turn regulates Nek2^24^. Aurora A is also required for centrosome separation, probably through its pivotal roles in regulating centrosome maturation and MT organization^25^.

Another group of kinases implicated in regulating mitotic events are the p21-activated kinases (Paks), which are well-known effectors of the Rac and Cdc42 GTPases^26^. Paks are frequently overexpressed in cancer and have numerous potentially oncogenic roles, including stimulating cell growth, survival and motility, which has led to the development of Pak inhibitors as potential therapeutics^27^. Paks fall into two structurally distinct groups, group I (Pak1-3) being activated directly on binding to active GTPases by a process involving autophosphorylation, and group II (Pak4-6) that are thought to be indirectly regulated by GTPases^28^. Among their many functions, group I Paks are known to be activated on the centrosomes in early mitosis^29^ and have been implicated in regulating mitotic entry^30^,^31^,^32^,^33^, consistent with their ability to phosphorylate Plk1 (ref. 31) and Aurora A^34^. However, the significance of the centrosomal group I Pak activity for mitotic progression has not been elucidated.

Here we advance our understanding of how the important process of centrosome separation is regulated. As well as identifying a novel Cdk1 site required for the function of Tiam1 in mitosis, we also show that group I Paks are activated downstream of Tiam1 on centrosomes in prophase in a manner dependent on the above phosphorylation event, and that both Pak1 and Pak2 function to antagonize centrosome separation. Importantly, we show that depletion of Pak1/2 confers resistance to mitotic arrest induced by Eg5 inhibitors, highlighting the potential importance of this pathway for cancer therapy.

## Results

### Tiam1 is phosphorylated in mitosis by Cdk1

**Figure 1:**
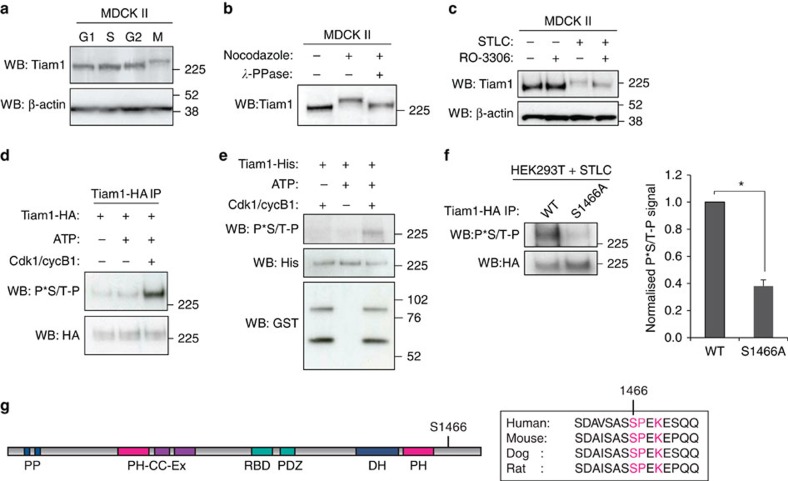
Cdk1-dependent phosphorylation of Tiam1 in mitosis. (**a**) MDCK II cells were synchronized in G1, S or G2 phases as described in Methods or in mitosis (M) by nocodazole treatment. Lysates were prepared and protein levels analysed by immunoblotting with the indicated antibodies. (**b**) Lysates were prepared from untreated or nocodazole treated MDCK II cells, treated with lambda phosphatase (*λ*-PPase) where indicated and analysed by immunoblotting. (**c**) MDCK II cells were left asynchronous or arrested in mitosis (STLC) then treated with RO-3306 (10 μM) where indicated for 3 h before lysis and immunoblotting. In **a** and **c** β-actin was used as a loading control. (**d**) Tiam1-HA was immunoprecipitated from HEK293T cells then subjected to *in vitro* kinase assay with ATP and GST-tagged Cdk1-cyclin B1 complex as indicated. Following SDS–PAGE, phosphorylation was measured by immunoblotting with anti-P*-Thr-Pro antibody (P*S/T-P). (**e**) Purified Tiam1-His was used for *in vitro* kinase assay with GST-tagged Cdk1-cyclin B1 and analysed as in **d**. (**f**) Tiam1-HA (either WT or the S1466A mutant) was immunoprecipitated from HEK293T cells arrested in mitosis (STLC) and analysed by immunoblotting with P*S/T-P antibody. Quantitation shows mean P*S/T-P normalized to HA signal+s.e.m. (with WT set as 1) (*n*=4, unpaired two-sided *t*-test: *P<0.05). (**g**) Schematic representation of Tiam1 protein showing the location of S1466, and conservation of this Cdk site in mammalian species. PP: PEST domains, PH-CC-Ex: Pleckstrin Homology, Coiled Coil, Extended region, RBD: Ras binding domain, PDZ: PDZ domain, DH: Dbl homology domain.

We and others have previously shown that Tiam1 can be regulated by phosphorylation^35^,^36^,^37^. To investigate whether phosphorylation might regulate Tiam1 during mitosis, we performed western blot analysis after synchronizing MDCK II cells at various stages of the cell cycle (Supplementary Fig. 1a). Our analysis revealed a shift in the mobility of Tiam1 when cells were arrested in mitosis with nocodazole compared with other cell cycle phases (Fig. 1a). Phosphatase treatment of lysates from mitotically arrested cells suppressed the mobility shift of Tiam1 (Fig. 1b). Moreover, pretreatment of cells with RO-3306, a Cdk1 inhibitor, suppressed the Tiam1 mobility shift of cells that had been arrested in mitosis using the Eg5 inhibitor STLC (Fig. 1c), suggesting that the chief mitotic kinase Cdk1 may be responsible for phosphorylation of Tiam1 in mitosis. We found that Cdk1, cyclin A and cyclin B1 all co-precipitated with Tiam1-GFP from asynchronous and mitotically arrested HEK293T cells (Supplementary Fig. 1b,c), supporting our hypothesis that Tiam1 interacts with active Cdk1 complexes. We used a Cdk/MAPK substrate-specific antibody that recognizes P*Ser/Thr-Pro to assess whether Cdk1 can phosphorylate Tiam1 *in vitro*. Kinase assays with a commercially obtained GST-tagged Cdk1–cyclin B1 complex and immunoprecipitated Tiam1 (Fig. 1d) or purified Tiam1-His (Fig. 1e) confirmed phosphorylation of Tiam1. We also found that Tiam1-His can be phosphorylated just as efficiently by Cdk1–cyclin A as with Cdk1–cyclin B1 (Supplementary Fig. 1d). Cdk1 is a proline-directed kinase that preferentially phosphorylates the consensus sequence S/T-P-X-K/R (where X is any amino acid), although it also phosphorylates the minimal sequence S/T-P^38^. Tiam1 contains 13 potential Cdk/MAPK sites (S/T-P) and one canonical Cdk1 site (S/T-P-X-K/R), S1466. We performed mass spectrometry analysis of TAP-tagged Tiam1 purified from mitotically arrested DLD1 cells and found that S1466 was phosphorylated (Supplementary Fig. 1e). We mutated S1466 in Tiam1-HA to alanine (S1466A) and analysed phosphorylation levels after immunoprecipitation from cells arrested in mitosis by STLC. This revealed a significant decrease in the Cdk phosphorylation signal of S1466A compared with wild-type Tiam1 (WT) (Fig. 1f), suggesting S1466 as a primary phosphorylation site for Cdk1 on Tiam1 in mitosis. S1466 is located to the C terminus of the catalytic DH-PH domain of Tiam1 and the Cdk1 consensus sequence is conserved in higher species (Fig. 1g), suggesting that this site may be important for Tiam1 function.

### S1466 phosphorylation by Cdk1 occurs on mitotic centrosomes

**Figure 2:**
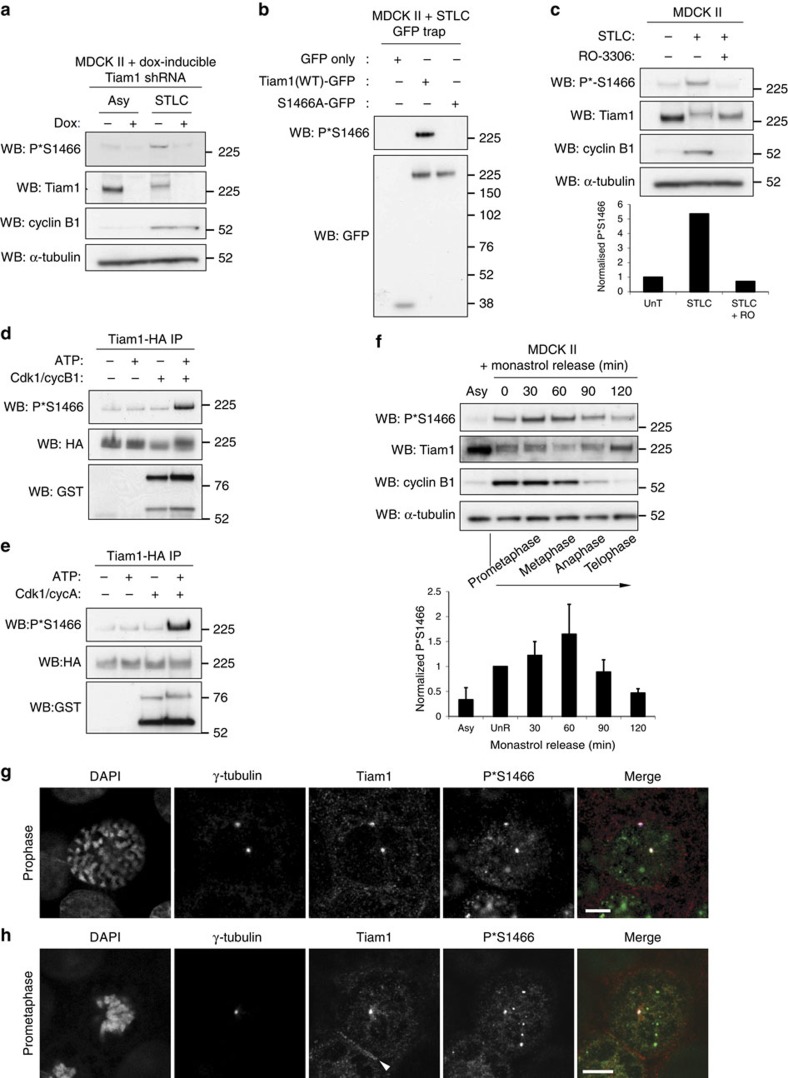
S1466 of Tiam1 is phosphorylated on centrosomes in early mitosis. (**a**) MDCK II cells expressing dox-inducible Tiam1 shRNA were grown for 6 days with or without dox as indicated, left asynchronous (Asy), or arrested in mitosis (STLC), lysed and analysed by immunoblotting with the indicated antibodies. (**b**) MDCK II cells stably expressing the indicated GFP-tagged constructs were arrested in mitosis with STLC and lysates subjected to GFP-trap, analysis by SDS–PAGE and immunoblotting with the indicated antibodies. (**c**) MDCK II cells were treated with STLC for 16 h, then Cdk1 inhibitor RO-3306 (RO) for 2 h as indicated, lysed and analysed by immunoblotting with the indicated antibodies. Graph shows quantitation of band intensities of P*S1446 normalized to total Tiam1 with untreated sample (UnT) set as 1. (**d**,**e**) Tiam1-HA was immunoprecipitated from HEK293T cells then subjected to *in vitro* kinase assay with addition of ATP and (**d**) GST-tagged Cdk1-cyclin B1 complex or (**e**) GST-tagged Cdk1-cyclin A complex where indicated. Phosphorylation was analysed by immunoblotting with an anti-P*S1466 antibody. (**f**) MDCK II cells were either left untreated (Asy) or treated for 16 h with monastrol (100 μM) to induce monopolar spindles, then released for the indicated times, lysed and analysed by immunoblotting with the indicated antibodies. Approximate mitotic stage for the time course is indicated. Graph shows mean P*S1466 normalized to total Tiam1 for the time course from three independent replicates+s.e.m. In (**a**,**c**,**f**) α-tubulin was used as a loading control. (**g**,**h**) MDCK II cells were fixed and stained by immunofluorescence (IF) with the indicated antibodies and DAPI. Merge: Tiam1=red, P*S1466=green, γ-tubulin=blue. Representative prophase (**g**) and prometaphase (**h**) cells are shown. White arrowhead indicates cell-cell junction. Scale bars, 5 μm.

To further investigate the temporal and spatial dynamics of the phosphorylation of Tiam1 on S1466, we raised an antibody against phosphorylated S1466. We demonstrated that this antibody recognized a band at the approximate size of Tiam1, which was stronger in lysates from cells arrested in mitosis with STLC and reduced on depletion of Tiam1 by RNAi (Fig. 2a). We also confirmed the specificity of the antibody by GFP-trap of WT and S1466A Tiam1-GFP from mitotically arrested cells followed by immunoblotting (Fig. 2b). We then treated mitotically arrested cells with RO-3306, which strongly reduced the P*S1466 signal for endogenous Tiam1 (Fig. 2c), indicating that phosphorylation at S1466 is dependent on Cdk1. Furthermore, a time course revealed reduced P*S1466 even within 5 min of RO-3306 treatment (Supplementary Fig. 2a). Performing kinase assays using Tiam1 immunoprecipitated from cells revealed that Cdk1 can indeed phosphorylate Tiam1 on S1466, both in complex with cyclin B1 (Fig. 2d) and cyclin A (Fig. 2e). To look more closely at the dynamics of S1466 phosphorylation, we arrested cells in a monopolar state using the Eg5 inhibitor monastrol, released them from this arrest and lysed at various time points as the cells progressed through mitosis. Phosphorylation on S1466 appeared to increase slightly following monastrol release, when the spindle was bipolarizing, then decreased following metaphase when cyclin B1 was degraded (Fig. 2f), consistent with a role in early mitosis. We also used the antibody to analyse the localization of phosphorylated endogenous Tiam1 by immunofluorescence. In mitotic cells, we saw a clear centrosomal signal that was most prominent in prophase (Fig. 2g). We confirmed that the centrosomal P*S1466 signal was specific as it was reduced in Tiam1-depleted cells (Supplementary Fig. 2b,c), and was present in WT , but not S1466A, Tiam1-GFP overexpressing MDCK II cells depleted for endogenous Tiam1 (Supplementary Fig. 2d). The P*S1466 signal could also be seen on centrosomes (as well as in other punctae) in prometaphase (Fig. 2h) and more weakly in metaphase and anaphase cells (Supplementary Fig. 2e). We observed co-localization of P*S1466 and total Tiam1 on centrosomes, whereas only total Tiam1 was present at cell-cell adhesions (Fig. 2h). These results show that Tiam1 is phosphorylated by Cdk1 on S1466, and the phosphorylated pool of Tiam1 is present on centrosomes in early mitosis.

### S1466 phosphorylation regulates centrosome separation

**Figure 3:**
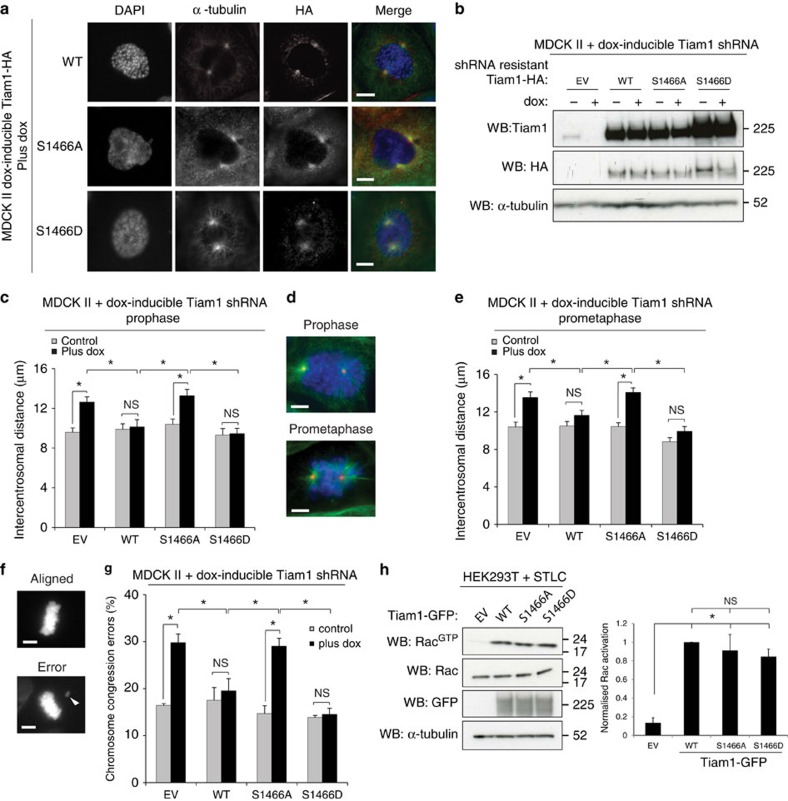
S1466 phosphorylation is required for regulation of centrosome separation and chromosome congression. (**a**) MDCK II cells expressing the indicated dox-inducible Tiam1-HA constructs were treated with dox for 4 days, fixed and stained by IF with the indicated antibodies. Merge: HA=red, α-tubulin=green, DAPI=blue. Representative prophase cells are shown. Scale bars, 5 μm. (**b**) MDCK II cells with dox-inducible Tiam1 shRNA and stable expression of the indicated HA-tagged Tiam1 constructs containing an RNAi-resistant mutation were treated with or without dox for 4 days, lysed and analysed by immunoblotting with the indicated antibodies. (**c–e**) The MDCK II rescue pools described in **b** were grown on coverslips without (control) or with dox (plus dox) for 4 days, fixed and stained by IF with antibodies against α-tubulin, γ-tubulin and DAPI. Intercentrosomal distances were measured for prophase (**c**) or prometaphase (**e**) cells (>50 cells over at least 3 independent experiments). (**d**) Example images for both phases. Prophase cells are defined as cells with condensed chromosomes but intact nuclear envelope. Prometaphase cells are cells after NEBD that have not yet aligned chromosomes to a clear metaphase plate. α-tubulin=green, γ-tubulin=red, DAPI=blue, scale bars: 5 μm. (**f**,**g**) The MDCK II rescue pools described in **b** were scored for chromosome congression errors (metaphase cells with clearly misaligned chromosomes as % of total metaphase cells). (**f**) Example DAPI images, scale bars, 5 μm, white arrowhead points to a misaligned chromosome. (**g**) Mean from three independent experiments (>60 cells/rep). (**h**) HEK293T cells transfected with the indicated GFP-tagged constructs were arrested in mitosis using STLC and subjected to Rac assay (top panel). Lower three panels show protein levels in input lysates. Graph shows quantification of Rac^GTP^/Rac for five independent replicates. All data show mean+s.e.m., unpaired two-sided t-test: *P<0.05, NS=not significant.

We next sought to determine whether phosphorylation of Tiam1 at S1466 was required for its function in mitosis. In addition to the non-phosphorylatable S1466A mutant, we mutated S1466 to aspartic acid (S1466D) to attempt to mimic phosphorylation and expressed HA-tagged forms of S1466A, S1466D and WT Tiam1 in MDCK II cells. Analysis of the localization of the S1466A and S1466D mutants in mitotic cells revealed a signal around mitotic asters, similar to the localization of WT Tiam1 (Fig. 3a, Supplementary Fig. 3a). Both mutants also localized to cell-cell adhesions to a similar extent to WT Tiam1 (Supplementary Fig. 3b), suggesting that Tiam1 localization is not affected by S1466 phosphorylation. To further assess the importance of S1466 phosphorylation for the mitotic functions of Tiam1, we re-expressed RNAi-resistant forms of WT, S1466A and S1466D Tiam1 into cells expressing dox-inducible Tiam1 RNAi (Fig. 3b). We then measured intercentrosomal distance in early mitosis and chromosome congression errors, both of which we have previously found to increase in Tiam1-depleted cells. We found that the S1466A mutant of Tiam1 was unable to rescue the increased intercentrosomal distance seen on Tiam1 depletion in both prophase (Fig. 3c,d) and prometaphase (Fig. 3d,e). The phospho-mimetic Tiam1 (S1466D), however, was able to rescue as effectively as WT Tiam1, suggesting that this mutation successfully mimics phosphorylation on this site. Scoring cells for chromosome congression errors similarly demonstrated that, whereas WT and S1466D Tiam1 rescued the increased chromosome congression errors seen on Tiam1 depletion, S1466A mutation was unable to rescue the phenotype (Fig. 3f,g). Although WT, S1466A and S1466D Tiam1-HA were overexpressed in these experiments, we saw no difference in cell morphology and no adverse effects on the later stages of mitosis with expression of S1466D Tiam1, suggesting that dephosphorylation of this site is not required for progression through the later stages of mitosis. To further substantiate the requirement for phosphorylation of Tiam1 on S1466 for centrosome separation we also made MDCK II cells with dox-inducible overexpression of RNAi-resistant HA-tagged WT, S1466A and S1466D Tiam1 and used a low level of dox to induce near-endogenous levels of exogenous Tiam1 (Supplementary Fig. 3c). We measured prophase intercentrosomal distance in these pools following transient transfection with Tiam1 siRNA, and found again that WT and S1466D Tiam1 could rescue the increased intercentrosomal distance seen on Tiam1 depletion, whereas S1466A was unable to rescue (Supplementary Fig. 3d).

Previously, we demonstrated that the ability of Tiam1 to regulate centrosome separation and chromosome congression was dependent on its Rac GEF activity^7^. We hypothesized that the failure of S1466A to effectively antagonize centrosome separation was due to an inability to activate Rac in mitosis. However, Rac assays following Tiam1 overexpression in mitotically arrested cells revealed similar activation of Rac by WT, S1466A and S1466D Tiam1 (Fig. 3h). We next wondered whether S1466 phosphorylation may alter the stability of Tiam1 in mitosis. To test this we generated MDCK II cells with dox-inducible WT or S1466A Tiam1 fused to a Halo-tag, which allows pulse-chase analysis of protein levels by ligand labelling^39^. However, no difference was found in the dynamics of Tiam1 degradation between WT and S1466A Tiam1 during the pulse-chase assay (Supplementary Fig. 3e). As S1466 creates a potential Polo-box binding site (S-S/T-P) when phosphorylated, and Plk1 plays multiple roles in regulating centrosome separation^20^,^22^,^24^, we also tested whether Plk1 interacts with Tiam1, and whether mutation of S1466 might affect this. Although we could clearly detect Plk1 co-precipitating with Tiam1-GFP in mitotically arrested cells, we saw no difference in the ability of S1466A or S1466D to interact with Plk1 compared with WT (Supplementary Fig. 3f). These results indicate that, although phosphorylation of S1466 does not appear to affect Tiam1 localization, stability or activity, it is essential to allow Tiam1 to regulate centrosome separation and chromosome congression.

### S1466 phosphorylation is required for Pak activation

**Figure 4:**
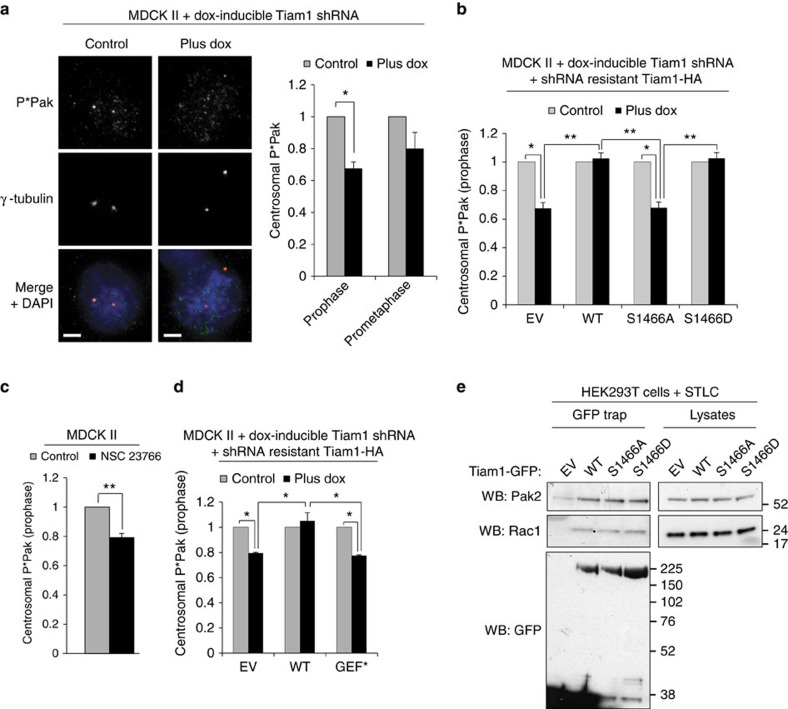
Group I Paks are activated on centrosomes downstream of Tiam1. (**a**) MDCK II cells with dox-inducible Tiam1 shRNA were grown for 4 days with (plus dox) or without dox (control) (for representative blot see Supplementary Fig. 4g), fixed and stained for IF. Merge: phospho-Pak1/2 (P*Pak)=green, γ-tubulin=red and DAPI=blue. Scale bars, 5 μm. Graph shows average centrosomal P*Pak levels for plus dox normalized to control in prophase (*n*=9) and prometaphase (*n*=5) (>40 centrosomes/rep). (**b**) MDCK II cell lines expressing dox-inducible Tiam1 shRNA and the indicated RNAi-resistant Tiam1 constructs were treated for 4 days with or without dox as indicated (blots for these cell lines are shown in Fig. 3b), fixed and stained for IF using antibodies against P*Pak1/2, γ-tubulin, HA and DAPI. Prophase cells with positive HA signal were analysed for centrosomal P*Pak as in **a** (*n*=4) (>40 centrosomes/rep). (**c**) MDCK II cells were control treated or treated with Rac1 inhibitor NSC 23766, fixed and stained for IF using antibodies against P*Pak1/2, γ-tubulin and DAPI. Centrosomal P*Pak in prophase cells was analysed as in **a** (*n*=3, >40 centrosomes/rep). (**d**) MDCK II cell lines expressing dox-inducible Tiam1 shRNA and the indicated RNAi-resistant Tiam1 constructs were treated for 4 days with or without dox as indicated (blots for these cell lines are shown in Supplementary Fig. 4d). Cells were fixed, stained and analysed for centrosomal P*Pak in prophase as in **b** (*n*=3, >40 centrosomes/rep). (**e**) HEK293T cells expressing GFP only (EV) or Tiam1-GFP (WT or phospho-mutant forms as indicated) were arrested in mitosis by STLC treatment, then lysates subjected to GFP-trap. Following SDS–PAGE, interaction with Pak2 and Rac was analysed by immunoblotting. All graphs show mean+s.e.m. Paired two-sided *t*-test: *P<0.05, **P<0.01.

As S1466 phosphorylation appears to be essential for the function of Tiam1 in mitosis, we next investigated whether this phospho-site would influence signalling downstream of Tiam1 and Rac. As the group I Paks, known Rac effector kinases, have previously been shown to be present on centrosomes in early mitosis, we hypothesized that in addition to their other mitotic roles^29^,^31^,^34^ Paks may also play a role downstream of Tiam1 and Rac in regulating centrosome separation. We used a phospho-Pak1/2 antibody (which detects autophosphorylation of Pak1 and Pak2) to detect Pak1/2 activity on the centrosomes of early mitotic cells. To test the specificity of the antibody we transfected MDCK II cells with siRNAs to either Pak1 or Pak2 (Supplementary Fig. 4a). We saw a large reduction in centrosomal P*Pak staining following knockdown of either Pak1 or Pak2 (shown for Pak1 siRNA; Supplementary Fig. 4b). We next analysed centrosomal P*Pak staining in MDCK II cells with dox-inducible Tiam1 RNAi and found that, on Tiam1 depletion, centrosomal phospho-Pak1/2 was significantly reduced by ∼35% in prophase (Fig. 4a). In prometaphase, phospho-Pak1/2 was also reduced though not as markedly as in prophase (20%, Fig. 4a). We next used sucrose density ultracentrifugation to isolate centrosomes from MDCK II cells with dox-inducible Tiam1 RNAi. In the fractions where the centrosomal marker γ-tubulin was the strongest, we also detected Tiam1 and P*Pak, and saw a decrease in P*Pak levels where Tiam1 was depleted by addition of dox (Supplementary Fig. 4c), supporting our immunofluorescence results that Tiam1 depletion decreases centrosomal Pak1/2 activity. To investigate the role of S1466 phosphorylation of Tiam1 in Pak activation, we measured centrosomal phospho-Pak1/2 in the MDCK II Tiam1 RNAi pools re-expressing RNAi-resistant phospho-mutant Tiam1 or WT Tiam1 (see Fig. 3b). Although WT and S1466D Tiam1 rescued the decrease in phospho-Pak1/2 seen on Tiam1 depletion in prophase, the S1466A mutant did not rescue the decrease (Fig. 4b), suggesting that phosphorylation on this site is required for centrosomal Pak1/2 activation in mitosis. As Pak1/2 are direct effectors of Rac1, we hypothesized that Tiam1-dependent Pak1/2 centrosomal activation was due to Tiam1 activation of Rac1. In agreement with this, MDCK II cells treated with the Rac1 inhibitor NSC 23766 had significantly decreased centrosomal P*Pak levels in prophase cells (Fig. 4c). Moreover, Tiam1 with a mutation in the Rac activation domain (GEF*), which renders it inactive, was unable to rescue the decrease in P*Pak seen on Tiam1 depletion (Fig. 4d, Supplementary Fig. 4d), strongly suggesting that regulation of centrosomal Pak1/2 activity requires Tiam1-mediated Rac activation. Interestingly, when we pulled down Tiam1-GFP from mitotically arrested cells, we detected co-precipitation with Rac, but also with both Pak2 (Fig. 4e) and Pak1 (Supplementary Fig. 4e). However, we saw no difference in the ability of the S1466A or S1466D mutant to co-precipitate Pak1 or Pak2 (Fig. 4e, Supplementary Fig. 4e), suggesting that phosphorylation of Tiam1 at S1466 is not required for its interaction with Pak1/2.

As Pak1 has been shown to phosphorylate Aurora A on T288^34^, and Aurora A could influence centrosome separation through its role in MT maturation and MT organization^25^, we also measured Aurora A activity on centrosomes using a P*T288 antibody (Supplementary Fig. 4f). However, phospho-Aurora A (P*Aurora A) levels at the centrosomes were unchanged in prophase following Tiam1 depletion (Supplementary Fig. 4g). We also measured centrosomal P*T288 of Aurora A after depleting Pak1 or Pak2 with two separate siRNA oligos to each Pak (Supplementary Fig. 4a), but found no difference in its staining intensity in prophase MDCK II cells (Supplementary Fig. 4h). These results suggest that Tiam1-mediated Rac activity is required for full activation of Pak1/2 on centrosomes in prophase, and that this change in Pak activity does not influence Aurora A activation. In addition, although S1466 phosphorylation of Tiam1 is not required for its binding to Pak1/2, it is required for their centrosomal activation.

### Pak1/2 regulate centrosome separation downstream of Tiam1

**Figure 5:**
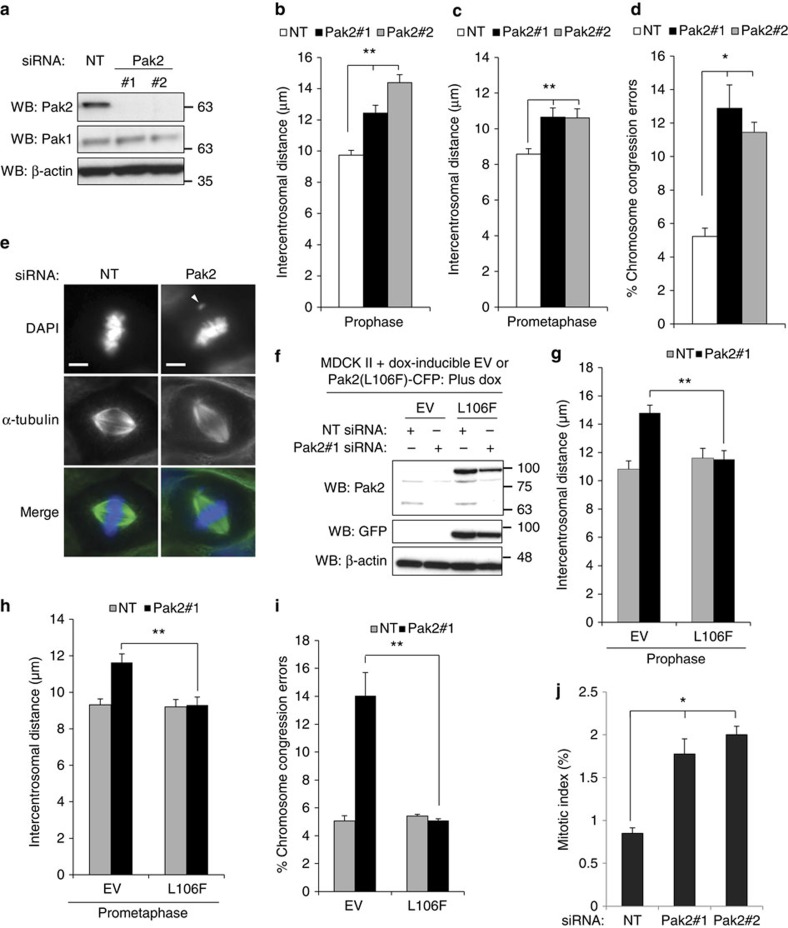
Group I Paks regulate centrosome separation. (**a**) MDCK II cells were transiently transfected with siRNAs against Pak2 or a non-targeting control (NT) as indicated and 2 days later lysed and analysed for knock-down by immunoblotting with the indicated antibodies. (**b**,**c**) Cells were transfected with siRNAs as in **a**, fixed and stained by IF with antibodies against α-tubulin, γ-tubulin and DAPI. Centrosomal distances in (**b**) prophase and (**c**) prometaphase were measured (>50 cells over at least three independent experiments). (**d**,**e**) Cells were transfected and stained as in (**b**,**c**) then analysed for chromosome congression errors (=metaphase cells with clearly misaligned chromosomes as % of total metaphase cells). (**d**) Quantification of chromosome congression errors from at least three independent replicates (>100 cells/rep). (**e**) Example IF images are shown. Merge: α-tubulin=green, DAPI=blue. White arrowhead indicates mis-aligned chromosome, scale bars, 5 μm. (**f**) MDCK II cells with dox-inducible overexpression of RNAi (Pak2#1)-resistant L106F (kinase active) Pak2-CFP or tet-ON construct only (EV) were transfected with NT or Pak2#1 siRNA and dox added 1 day later for 1 day before lysis and analysis by immunoblotting with the indicated antibodies. (**g**,**h**) Cells treated as in **f** were fixed and stained for IF with antibodies against GFP, γ-tubulin and DAPI. Centrosomal distances in (**g**) prophase and (**h**) prometaphase were measured for cells expressing the EV or Pak2(L106F)-CFP constructs (as determined by presence of positive staining with GFP antibody) >60 cells over three independent experiments). (**i**) Cells treated and stained as in (**f**–**h**) were scored for chromosome congression errors (in cells positive for GFP signal) (*n*=3, >50 cells/replicate). (**j**) MDCK II cells were transiently transfected with the indicated Pak2 siRNAs, then 2 days later fixed and stained for FACS analysis with MPM2 antibody and propidium iodide. % MPM2 positive cells in the G2 peak represent mitotic index (mean+s.e.m., *n*⩾3, 10,000 cells/rep). All graphs show mean+s.e.m. Unpaired two-sided *t*-test: *P<0.05, **P<0.001.

We next determined whether Pak is required for regulation of centrosome separation as we have found for Tiam1 and Rac. Following depletion of either Pak1 or Pak2 in MDCK II cells using two sets of siRNA oligos (Fig. 5a and Supplementary Fig. 5a), we measured intercentrosomal distance in prophase and prometaphase. We found that depletion of either Pak1 (Supplementary Fig. 5b,c) or Pak2 (Fig. 5b,c) significantly increased the intercentrosomal distance at both phases and with both oligos. We also assessed the ability of cells to effectively align their chromosomes to the metaphase plate and found that depletion of either Pak1 (Supplementary Fig. 5d) or Pak2 (Fig. 5d,e) increased the proportion of chromosome congression defects. To confirm that the effects we measured were due to depletion of Pak rather than off-target effects of the siRNAs, we generated MDCK II cells with dox-inducible expression of active (L106F) Pak2-CFP with mutations rendering it resistant to Pak2#1 siRNA (Fig. 5f). In the control cells, we again saw an increase in intercentrosomal distance in both prophase (Fig. 5g) and prometaphase (Fig. 5h), as well as increased chromosome congression errors (Fig. 5i) when we transfected with Pak2#1 siRNA. However, induction of Pak2(L106F)–CFP completely rescued all three phenotypes (Fig. 5g–i). Moreover, the mitotic index in Pak1/2-depleted cells was significantly increased by ∼twofold with both oligos to Pak1 (Supplementary Fig. 5e) and Pak2 (Fig. 5j), consistent with cells requiring longer to progress through mitosis as we have seen previously following Tiam1 depletion^7^. We also quantified cell cycle stages by FACS analysis following Pak depletion but found no significant difference in the proportion of G1, S or G2 cells for any of the Pak siRNA oligos compared with a non-target control (Supplementary Fig. 5f). This suggests that individual depletion of Pak1 or Pak2 does not have a major effect on cell-cycle progression in MDCK II cells, but that they act to antagonize centrosome separation and are consequently required for normal mitotic progression.

**Figure 6:**
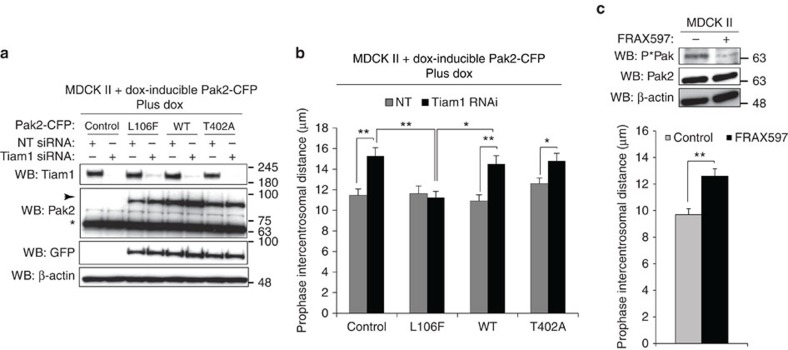
Pak activity is required for regulation of centrosome separation downstream of Tiam1. (**a**,**b**) MDCK II cells with dox-inducible overexpression of WT, L106F (kinase active) or T402A (kinase inactive) Pak2-CFP or tet-ON construct only (Control) were transfected with non-targeting control (NT) or Tiam1 siRNA and dox added 1 day later for 1 day. (**a**) Cells were lysed and analysed by immunoblotting with the indicated antibodies. Arrow indicates expression of exogenous Pak2-CFP constructs; *endogenous Pak2. (**b**) Cells were stained for IF with antibodies against GFP, γ-tubulin and DAPI, and intercentrosomal distance of cells in prophase expressing the different Pak2-CFP constructs (as determined by presence of positive staining with GFP antibody, see Supplementary Fig. 6b) was measured (>50 cells over three independent experiments). (**c**) MDCK II cells were treated for 6 h with 2 μM FRAX597 and either lysed and analysed by immunoblotting for Pak activity (P*Pak) or fixed and stained for IF with antibodies against α-tubulin, γ-tubulin and DAPI then intercentrosomal distance in prophase measured (>50 cells over three independent experiments). All graphs show mean+s.e.m. Unpaired two-sided *t*-test: *P<0.05, **P<0.001.

To address whether group I Paks function specifically downstream of Tiam1 in regulating centrosome separation, we generated MDCK II cells with stable dox-inducible expression of either WT, kinase inactive (T402A) or kinase active (L106F) Pak2-CFP, and confirmed their relative activities using P*Pak antibody (Supplementary Fig. 6a). To assess the impact of differentially activated Pak2 on the Tiam1-dependent mitotic phenotype we transiently depleted Tiam1 while also inducing expression of Pak2-CFP in these cell lines (Fig. 6a). Interestingly, we found that kinase active L106F Pak2-CFP completely suppressed the increased intercentrosomal distance seen on Tiam1 depletion in prophase, whereas WT Pak2-CFP and kinase-dead T402A Pak2-CFP failed to suppress the increase (Fig. 6b), despite all three forms of Pak2-CFP showing similar expression levels and localization (Fig. 6a and Supplementary Fig. 6b). In addition, treatment with FRAX597, an inhibitor of group I Paks^40^, increased intercentrosomal distance in prophase (Fig. 6c). These results strongly suggest that group I Paks function downstream of Tiam1 to oppose centrosome separation in early mitosis, and that this function requires their kinase activity.

We wanted to investigate whether the regulation of centrosome separation by the Tiam1-Pak-Rac signalling pathway was also operating in cells other than MDCK II. Depletion of Tiam1 or Pak2 in U2OS (human osteosarcoma) cells also led to increased intercentrosomal distance in prophase (Supplementary Fig. 6c,d). In addition, we saw increased P*S1466 signal by immunoblot in lysates from mitotically arrested U2OS cells, which was reduced on Cdk1 inhibitor treatment (Supplementary Fig. 6e). We also made U2OS cells with dox-inducible expression of the S1466D and S1466A forms of Tiam1 (Supplementary Fig. 6f). Expression of the S1466D, but not the S1466A mutant, rescued the increased prophase intercentrosomal distance following depletion of Tiam1 by RNAi (Supplementary Fig. 6g). These findings show that the signalling pathway we have identified is also important in other systems.

### Pak signalling modulates responses to Eg5 inhibitors

**Figure 7:**
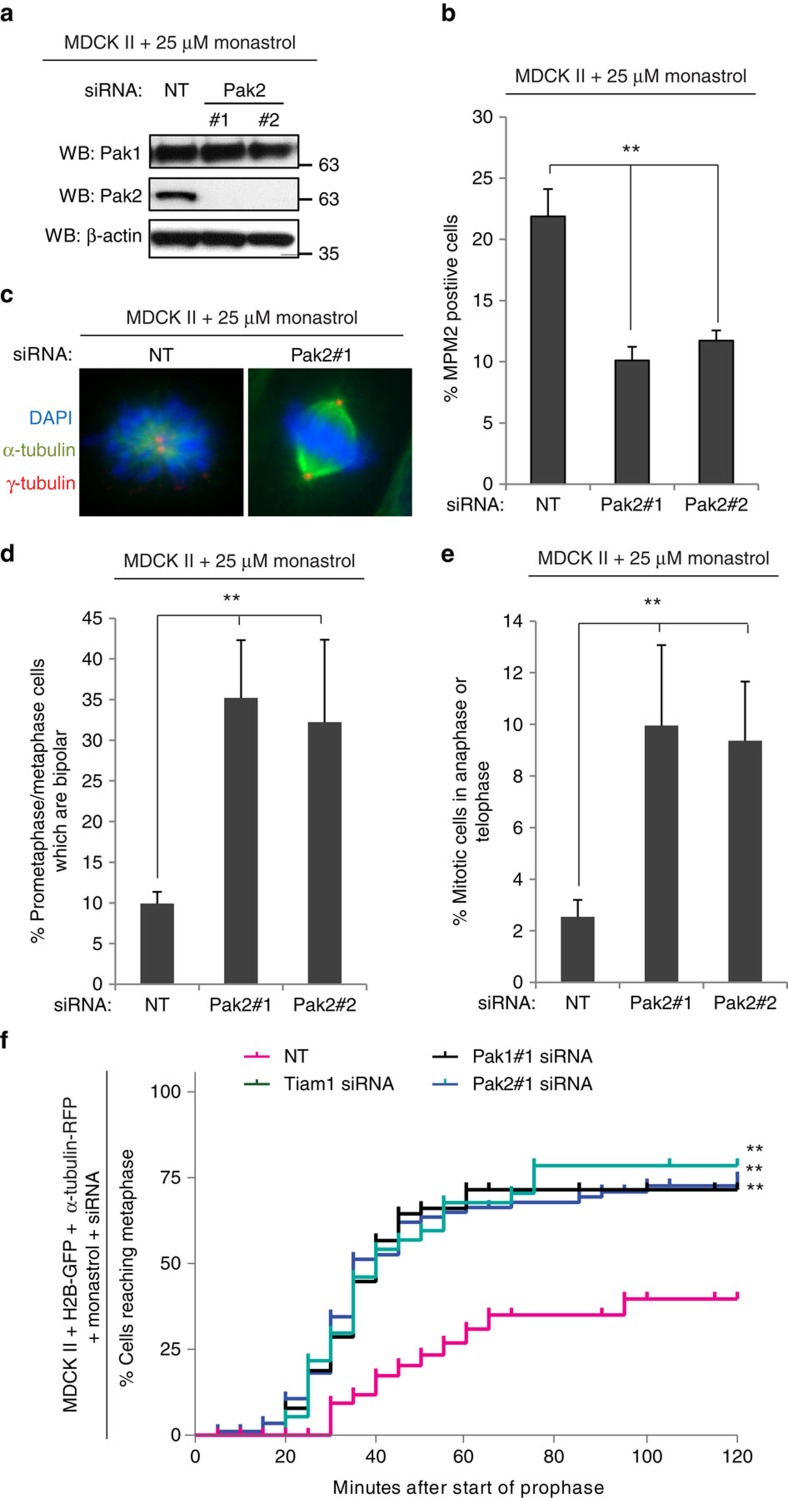
Tiam1 and Pak modulate responses to Eg5 inhibitors. (**a–e**) MDCK II cells were transiently transfected with two siRNAs against Pak2 or non-targeting control (NT) as indicated, and 2 days later treated with 25 μM monastrol for 16 h. (**a**) Cells were lysed and analysed for knockdown by immunoblotting with the indicated antibodies. (**b**) Cells were fixed and stained for FACS analysis with propidium iodide and MPM2 antibody to detect % of mitotic cells (*n*⩾3, 10,000 cells/rep). (**c–e**) Cells were fixed and then stained by IF with antibodies against α-tubulin, γ-tubulin and DAPI. (**c**) Example mitotic spindles: α-tubulin=green, γ-tubulin=red and DAPI=blue, scale bars, 5 μm. (**d**) Quantification of the proportion of bipolar spindles (*n*⩾3). (**e**) Quantification of the proportion of mitotic cells in anaphase and telophase (*n*⩾3). (**b–e**) All graphs show mean+s.e.m. Unpaired two-sided *t*-test: **P<0.01. (**f**) MDCK II cells expressing histone-2B-GFP (H2B-GFP) and α-tubulin-RFP were transiently transfected with siRNAs against Tiam1, Pak1, Pak2 or non-targeting control (NT) as indicated for 2 days then treated with 25 μm monastrol before being imaged using time-lapse confocal microscopy as described in Methods. Graph shows % of cells which reached metaphase as a function of time after the start of prophase (first frame with evidence of condensed chromosomes). Small vertical lines indicate cells at the end of imaging time. Gehan-Breslow-Wilcoxon test: **P<0.001 (compared with NT control).

As Pak1 and Pak2 function to antagonize centrosome separation downstream of Tiam1 we sought to determine whether Pak1/2 knockdown allows cells to escape monopolar arrest by Eg5 inhibitors, as we have seen previously with Tiam1 depletion^7^. For this, we treated MDCK II cells transfected with non-targeting (NT) or Pak1/2 siRNAs (Fig. 7a, Supplementary Fig. 7a) with an intermediate concentration (25 μM) of the Eg5 inhibitor monastrol and analysed mitotic index by FACS analysis. A significant decrease in mitotic index was detected with both oligos for Pak1 (Supplementary Fig. 7b) and Pak2 (Fig. 7b). To determine whether this was due to effects on mitotic spindle assembly, we assessed the mitotic stage of the above cells. We observed a significant increase in both the proportion of bipolar spindles (Fig. 7c,d, Supplementary Fig. 7c), and the proportion of cells in anaphase and telophase (Fig. 7e, Supplementary Fig. 7d) in cells depleted of Pak1 or Pak2. Further, by live imaging of prophase MDCK II cells expressing histone-2B-GFP and α-tubulin-RFP we verified that after treatment with siRNA to Pak1, Pak2 or Tiam1 the majority of monastrol treated cells entered metaphase, whereas the majority of control cells arrested in prometaphase (Fig. 7f, Supplementary Fig. 7e, Supplementary Movies 1–4). This shows that, like Tiam1-Rac signalling, levels of Pak1 and Pak2 alter the sensitivity of cells to Eg5 inhibitor, highlighting the importance of this signalling pathway for clinical studies with Eg5 inhibitors.

## Discussion

Our study provides significant insights into the regulation of the important process of centrosome separation. First, we find that Cdk1 phosphorylation of Tiam1 on a highly conserved canonical phosphorylation site, S1466, is required for Tiam1-dependent antagonism of centrosome separation. Although Cdk1 is central to the regulation of mitotic entry and phosphorylates Eg5 to stimulate its binding to MTs^21^, centrosome separation can still occur under Cdk1 inhibition, though with slower dynamics^22^. Our results shed new light onto the role of Cdk1 in centrosome separation, by demonstrating that Cdk1 lies upstream of pathways that both drive and oppose centrosome separation to maintain the correct balance for efficient chromosome alignment.

This work also describes a novel function of Pak1/2 in regulating centrosome separation, which, despite the increasing interest in this group of kinases^27^, had not previously been identified. Group I Paks are known to be active on mitotic centrosomes^29^ and a few studies report that Pak1/2 contribute to proper timing of the G2/M transition^30^,^31^,^32^,^33^. This is consistent with their ability to phosphorylate Plk1 (ref. 31) and Aurora A^34^, though the contribution of individual Paks to regulating entry into mitosis, the mechanism of Pak1/2 centrosomal activation, and how Paks then regulate mitotic entry are all unclear. It has been shown that Pak1 can be activated on centrosomes independently of GTPases by a mechanism mediated by GIT1-PIX^34^. However, a recent study implicated Rac upstream of centrosomal Pak2 activation and Aurora A activation in early mitosis^30^. Therefore, it is possible that multiple, potentially redundant, pathways activate Pak1/2 to promote mitotic entry. While our data support the inference that Pak activation at centrosomes is downstream of Rac (Fig. 4c,d), we showed that phosphorylation of Aurora A is independent of Tiam1-Rac-Pak signalling, at least during prophase (neither knockdown of Tiam1, nor of individual Pak isoforms—which perturb centrosome separation—appeared to change the phosphorylation of Aurora A; Supplementary Fig. 4f–h). As Aurora A can promote centrosome separation^41^,^42^,^43^, we would not have anticipated activation of Aurora A by Tiam1-Rac-Pak signalling, which we demonstrate antagonizes centrosome separation in prophase. However, we cannot exclude that Aurora A may be affected by Pak knockdown at other temporal stages, or whether both Pak1 and Pak2 need to be depleted to see an effect on Aurora A activity.

Through re-expression of phospho-mutant and GEF mutant Tiam1 we showed that activation of Pak1/2 at the centrosomes in prophase is dependent both on phosphorylation of Tiam1 at S1466 (Fig. 4b) and its ability to activate Rac (Fig. 4d). As we saw no difference in the localization, activity or stability of S1466A Tiam1 compared with WT (Fig. 3a,h, Supplementary Fig. 3a,e) we speculate that S1466 phosphorylation may be involved in a scaffolding function of Tiam1 at the centrosomes. Tiam1 has previously been shown to direct Rac signalling by binding to other proteins^44^,^45^. While S1466 phosphorylation does not appear to affect binding of Tiam1 to Pak1/2 (Fig. 4e, Supplementary Fig. 4e), it may be required to mediate activation of Pak1/2 by Rac potentially through recruitment of other as yet unknown members of the centrosomal signalling complex. Future efforts will focus on identifying these factors. Such endeavour may also identify Pak1/2 substrates required to antagonize centrosome separation. These could be proteins that regulate MT stability, as MT stability has been shown to influence centrosome separation and sensitivity to Eg5 inhibitors^7^,^22^,^46^. Interestingly, a number of Pak substrates have been described that could influence MT function including MCAK^47^, TCoB^48^, stathmin^49^ and GEF-H1 (ref. 50). Tiam1 is also localized to the cortex where it could potentially influence centrosome separation through astral-MT interactions with the cortex. However, the fact that P*S1466 is detected at centrosomes but not cell-cell adhesions (Fig. 2h) indicates that the P*Tiam1-Rac-Pak complex acts from the centrosomes.

Both Tiam1 (ref. 14) and Pak1 (ref. 51) are required for Ras-induced tumorigenesis *in vivo*. Although the function we have described here for Tiam1-Pak-Rac signalling could contribute to cell growth through promoting mitotic progression, perhaps more important is the possibility that loss of Tiam1 or Pak1/2, or de-regulation of Tiam1 S1466 phosphorylation could lead to chromosome mis-segregation, which could in turn promote malignant progression. Intriguingly, the few Ras-induced skin tumours that form in *Tiam1*-knockout mice are more frequently malignant than in control mice^14^, and Tiam1 protein expression is decreased during breast cancer progression^52^. Although this could be due to the role of Tiam1 in regulating cell-cell adhesions^53^, CIN could also be a contributing factor. Paks are very frequently amplified, overexpressed or hyperactivated in cancers, and contribute positively to proliferation through a number of mechanisms, including stimulation of the Ras/Raf/MEK/Erk pathway^27^. The interest in Paks as potential therapeutic targets has led to the development of Pak inhibitors, which have shown promising results for *in vivo* models of melanoma^54^ and Ras-induced skin tumours^51^. Whether reduction of Pak1/2 activity can lead to increased tumour aggressiveness remains to be addressed, but as the function of Pak1/2 downstream of Tiam1 is kinase dependent (Fig. 6), a consideration for any future Pak inhibitor trials is that treatment could lead to mitotic defects and potentially CIN through the pathway we have identified.

This study also has clear implications for the ongoing clinical trials of Eg5 inhibitors for cancer therapy^10^. We have shown that depletion of Pak1/2 affects sensitivity to the Eg5 inhibitor monastrol (Fig. 7 and Supplementary Fig. 7), as we have seen previously for Tiam1 depletion^7^. These results suggest that modulation of the Tiam1-Rac-Pak signalling pathway could potentially confer resistance to Eg5 inhibitors and highlights that further study of this pathway will be important to inform future clinical studies with this class of drugs.

## Methods

### Antibodies

Working dilutions of antibodies for immunoblotting (IB), immunofluorescence (IF) and flow cytometry (FC) are shown below. IP indicates used for immunoprecipitation. Anti-Tiam1 antibodies: rabbit (IB 1:1,000, Bethyl Laboratories, A300-099A) and sheep (IF 1:200, R&D Systems, AF5038), anti-β-actin mAb (IB 1:10,000, AC15, Sigma, A5441), anti-Cdk1 (IB 1:1,000, Cell Signalling, #2655), anti-cyclin B1 (IB 1:1,000, Cell Signalling, #4138), anti-cyclin A mAb (IB 1:1,000, E67.1, Santa Cruz, sc-53230), anti-GFP (IB 1:10,000; IF 1:500, Abcam, ab290), anti-phospho-Thr-Pro mAb (‘P*S/T-P’) (IB 1:5,000, P-Thr-Pro-101, Cell Signalling, #9391), anti-6xHis mAb (1:10,000, Clontech, 631212), anti-P*S1466 (Tiam1) (IB 1:1,000, IF 1:1,000, custom-made by Eurogentec), anti-α-tubulin (IB 1:5000; IF 1:2,500, DM1A, Sigma, T9026), Anti-HaloTag mAb (IB 1:1,000, Promega, G9211), anti-Rac1 (IB 1:1,000, BD, 610650), anti-Plk1 (IB 1:2,000, Upstate, #06-813), anti-HA mAb (IB 1:10,000; IP, 12CA5, Roche, 11583816001), anti-HA (IB 1:10,000; IF 1:200; IP, AbCam, ab9110), anti-γ-tubulin antibodies: rabbit (IF 1:2,000, Sigma, T5192), and mouse mAb (IF 1:5,000, GTU-88, AbCam, ab11316), anti-centrin mAb (IF 1:2,000, 20H5, Millipore, 04-1624), phospho-T288-Aurora A (IF: 1:1,000, AbCam, ab83968), anti-Pak(1/2/3) (IB 1:2,000, Cell Signalling, 2604), anti-phospho-Pak1/2 [P*Pak1 (S199/204), P*Pak2(S192/197), IB 1:1,000, IF 1:500, Cell Signalling, #2605], anti-Pak1 (IB 1:2,000, Cell Signalling, #2602), anti-Pak2 mAb (IB 1:2,000, C17A10, Cell Signalling, #2615), anti-phospho-Ser/Thr-Pro mAb (FC 1:2,000, MPM2, Upstate, 05-368MG), HRP-conjugated anti-GST (IB 1:10,000, AbCam, ab3416).

Secondary antibodies: IgG-peroxidase-conjugated (IB 1:5,000, GE Healthcare), Alexa Fluor 488, 568, 647-conjugated (IF 1:500, Molecular Probes), APC-conjugated (FC 1:1,000, Molecular Probes). Full scans of gels can be found at the end of the Supplementary Information.

### Constructs

The following constructs containing full-length (FL) mouse Tiam1 cDNA (GenBank accession NM_009384) have previously been described: pCDNA3-Tiam1-HA^36^,^55^,^56^, Tiam1-HA-CTAP^57^, pRetro-XT-Tiam1-HA(puro)^56^, Tiam1-HA-IRES-DsRed (EV, WT and GEF*) (containing an RNAi-resistant Tiam1 sequence^7^) and pBOS-Histone-2B-GFP^7^. pEGFP-Tiam1-HA was made in-house by cloning Tiam1-HA from pCDNA3-Tiam1-HA using NdeI-FseI sites. pRetro-XT-Tiam1-Halo(puro) was made by insertion of the Halo tag amplified from pFC14A (Promega) into pRetro-XT-Tiam1-HA(puro). pRetro-XT-Tiam1-HA-GFP(puro) was made by subcloning the eGFP tag fused to Tiam1 in pEGFP-Tiam1-HA into pRetro-XT-Tiam1-HA(puro). Phospho-mutant forms of pcDNA3-Tiam1-HA, pEGFP-Tiam1-HA, pRetro-XT-Tiam1-HA(puro), pRetro-XT-Tiam1(Halo)(puro), pRetro-XT-Tiam1-HA-GFP(puro) and Tiam1-HA-IRES-DsRed were generated by site-directed mutagenesis using QuikChange (Stratagene) according to the manufacturer’s instructions. Generation of the single-vector tetracycline-repressor-based inducible shRNA system for Tiam1 has been described previously^7^, the specific target RNAi sequence is 5′- GAGGTTGCAGATCTGAGCA -3′. Pak2(WT)-CFP was kindly provided by Claire Wells (King’s College, London), and T402A and L107F versions of Pak2-CFP were generated by mutagenesis of this construct. pRetro-XT-PAK2-CFP(puro) (WT, T402A and L107F) were made by subcloning from Pak2-CFP (WT, T402A and L107F) to pRetro-X-tight(puro) (Clontech) using NruI/NotI sites. pRetro-X-tight(puro)-Pak2(L106F)-CFP with RNAi-resistant mutations was produced by site-directed mutagenesis. FL Tiam1 (WT) Bac-to-Bac CT-TOPO was generated in-house by subcloning FL Tiam1 (WT) from pCDNA3-Tiam1-HA into the pFAST BAC/CT-TOPO (Invitrogen). pRetro-Tet-ON and pEGFP constructs were from Clontech, and pmRFP-α-tubulin-C1 was from AddGene (plasmid # 21040).

### Cell culture

All cells were cultured in a 37 °C, 5% CO_2_ incubator. Parental MDCK II, U2OS and HEK293T cells (from ECACC, operated by Public Health England) were maintained in Dulbecco’s Modified Eagle Medium (DMEM, Invitrogen) in the presence of 10% fetal bovine serum (FBS, GIBCO). Cell lines were routinely tested for mycoplasma contamination by our in-house facility. DLD1 cells expressing Tiam1-HA-CTAP were maintained in the same medium supplemented with Zeocin (0.25 mg ml^−1^, Invitrogen). MDCK II with dox-inducible Tiam1 shRNA and re-expressing Tiam1-HA-IRES-DsRed were maintained in DMEM with 10% tetracycline-free FBS (Autogen Bioclear) and G418 (1 mg ml^−1^, Sigma). MDCK II with dox-inducible Tiam1-HA, Tiam1-GFP, Tiam1-Halo and Pak2-CFP and U2OS with dox-inducible Tiam1-HA were maintained in DMEM with 10% tetracycline-free FBS with G418 and puromycin (2 μg ml^−1^, Sigma). MDCK II cells expressing histone-2B-GFP and α-tubulin –RFP were grown in DMEM with 10% tetracycline-free FBS with G418 and blasticidin (5 μg ml^−1^, Sigma).

### Generation of cell lines

Plasmids were introduced into cells either by transfection using TransIT-LT1 (Mirus) according to the manufacturer’s instructions or by retroviral transduction as previously described^36^. For MDCK II cells stably expressing GFP and Tiam1-GFP (WT, S1466A and S1466D), transfection was followed by selection in G418 (1 mg ml^−1^, Sigma) and sorting for GFP-positive cells. For inducible overexpression, MDCK II or U2OS cells were retrovirally transduced with pRetro-Tet-ON followed by selection with G418. pRetro-XT-based constructs were then retrovirally transduced and cells selected with puromycin (2 μg ml^−1^, Sigma). To obtain cells expressing RNAi-resistant Tiam1 (where the selection marker was the same as the target Tiam1 RNAi MDCK II cells), the various Tiam1-HA-IRES-DsRed constructs were retrovirally transduced and DsRed-positive cells were FACS-sorted for further analysis. MDCK II cells expressing histone-2B-GFP and α-tubulin-RFP were made by first transfecting with pBOS-Histone-2B-GFP following by selection in blasticidin (5 μg ml^−1^, Sigma). pmRFP-α-tubulin-C1 was then transfected, cells selected with G418 and sorted for GFP/RFP double positives.

### Cell treatments

To synchronize in G1, cells were placed in serum-free media for 24 h. To arrest cells in S phase, cells were incubated for 16 h with 2 mM thymidine, released by washing three times with PBS and placed into fresh medium for 6 h, followed by a second incubation with 2 mM thymidine for 16 h. Cells in the G2 phase of the cell cycle were obtained 4 h after release from double thymidine block. Where nocodazole was used to arrest cells in mitosis, treatment was with 50 μg ml^−1^ nocodazole (Sigma) for 16 h. Where Eg5 inhibitor was used to arrest cells in mitosis, cells were incubated with S-trityl-L-cysteine (STLC, Sigma, 5 μM) or monastrol (Tocris, 100 μM) for 16 h unless otherwise stated. Release from monastrol was achieved by washing cells three times in PBS and replacing media. Cdk1 inhibitor (RO-3306, Alexis) was used at 10 μM. Treatment with the Pak inhibitor FRAX597 (a kind gift from Jonathan Chernoff, Fox Chase Cancer Center, Philadelphia, USA) was at 2 μM for 6 h, and with NSC 23766 for 24 h at 100 μM. For dox-inducible expression of shRNA or Pak2-CFP or Tiam1-HA constructs, cells were treated with doxycyline (1 μg μl^−1^) for 3–4 days (shRNA), or 24 h (Pak2-CFP, Tiam1-HA constructs).

### Transient transfection of plasmids and siRNA

Plasmids were introduced into HEK293T by transient transfection using TransIT-LT1 (Mirus) according to the manufacturer’s instructions and processed after at least 24 h. Transient silencing of Pak1, Pak2 and Tiam1 was achieved by transfection of siRNA oligos from Eurofins MWG operon into MDCK II or U2OS cells using Lipofectamine RNAiMax (Invitrogen, Life Technologies) according to the manufacturer’s instructions. Cells were processed and analysed 48 h post transfection. siRNA sequences were as follows:

Pak1#1: 5′- GGATGCGGCTACATCTCCTATTTCA -3′

Pak1#2: 5′- CAGCCGAAGAAAGAGCTGATTATTA -3′

Pak2#1: 5′- CAGAGGTGGTTACACGGAAAGCTTA -3′

Pak2#2: 5′- GCCAGAACAGTGGGCTCGATTATTA -3′

Tiam1 RNAi: 5′- GAGGTTGCAGATCTGAGCA -3′

Two non-targeting control oligos were used: Dharmacon—non-targetting siRNA #4 and HNT: 5′- AGGTAGTGTAATCGCCTTG -3′.

### Protein analysis

Cells were lysed in IP lysis buffer (50 mM Tris-HCl, pH 7.5, 150 mM NaCl, 1% (v/v) Triton-X-100, 10% (v/v) glycerol, 2 mM EDTA, 25 mM NaF and 2 mM NaH_2_PO_4_) containing a protease inhibitor cocktail (Sigma) and phosphatase inhibitor cocktails 1 and 2 (Sigma). For immunoprecipitation, lysates were incubated with 1 μg anti-HA antibody pre-bound to 20 μl of GammaBind G Sepharose (Amersham) for 2 h at 4 °C, washed in IP lysis buffer, then eluted with 1 × SDS–PAGE sample buffer (Nupage, Invitrogen). GFP-trap was similarly performed by incubating lysates with 10–20 μl GFP-trap beads (Chromotek) for 45–60 min before washes and addition of SDS sample buffer. Treatment of cell lysates with lambda phosphatase (Biolabs) was for 30 min at 30 °C. Samples in SDS sample buffer were resolved by SDS–PAGE and transferred to PVDF membrane (Immobilon-P; Millipore). Immunoblotting was performed according to the protocol for specific primary antibodies. Band intensities were quantified using Genetools (SynGene). To measure endogenous Rac GTPase activity, cells were lysed on ice in GST-FISH buffer (50 mMTris-HCl, pH 7.5, 100 mM NaCl, 2 mM MgCl_2_, 1% (v/v) Nonidet P-40, 10% (v/v) glycerol and protease inhibitors (Complete, EDTA-free; Roche)) containing 1 μg of a biotinylated PAK-derived CRIB (Cdc42/Rac interacting binding) peptide (custom made; CRUK Protein and Peptide Chemistry) per assay. Cleared cell lysates were incubated at 4 °C for 20 min; active Rac/CRIB complexes were precipitated using streptavidin-conjugated agarose beads (Sigma) for a further 15 min at 4 °C. Following washes in GST-FISH buffer, protein samples were eluted with 1 × SDS–PAGE sample buffer, and processed for western blotting with anti-Rac antibody as above.

### TAP purification and mass spectrometry

Nocodazole-treated DLD1 cells expressing Tiam1-HA-CTAP were lysed in IP lysis buffer and TAP purification performed as previously described^57^. In brief, lysates of cells were prepared on ice in IP lysis buffer and cleared by centrifugation for 20 min; this and all subsequent steps were performed at 4 °C. Cleared lysates were incubated with IgG sepharose beads (GE Healthcare) for 2 h before washing four times in TAP wash buffer (50 mM Tris-HCl, pH 7.5, 150 mM NaCl, 0.1% (v/v) Triton-X-100, 0.5 mM EDTA and 1 mM b-mercaptoethanol), and once in protease cleavage buffer (50 mM Tris-HCl, pH 7.5, 150 mM NaCl, 0.1% (v/v) Triton-X-100 and 1 mM DTT). Protein complexes were eluted from IgG beads by incubating with Tobacco Etch Virus (AcTEV) protease (Invitrogen) in 1 ml of protease cleavage buffer for 2 h. The eluate was diluted one in six with calmodulin binding buffer (CBB; 50 mM Tris-HCl, pH 7.5, 150 mM NaCl, 0.1% (v/v) Triton-X-100, 1 mM MgCl_2_, 1 mM imidazole, 4 mM CaCl_2_ and 10 mM b-mercaptoethanol) before adding to Calmodulin affinity resin (Stratagene), prewashed in CBB and incubated for 1 h. After incubation, samples were washed four times with CBB, eluted with 1 × SDS–PAGE sample buffer and resolved by SDS–PAGE (4–12% Nupage gel; Invitrogen). Gels were stained using coomassie (SimplyBlue Safe stain; Invitrogen) and the Tiam1-TAP band excised, destained and tryptically digested. LC MS/MS to identify Tiam1 phosphopeptides was performed using a MIDAS workflow^58^ on a 4,000 Q-TRAP tandem mass spectrometer (AB SCIEX). Potential sites of phosphorylation were confirmed using Mascot (Matrix Science) and *de novo* sequencing.

### Purification of Tiam1-His from insect cells

Full-length Tiam1 was expressed as a poly-His-fusion protein in Sf9 insect cells according to the protocol from Bac-to-Bac TOPO expression system (Invitrogen). Sf9 cells were pelleted and lysed in Buffer A (50 mM Tris-HCl pH 8.0, 5 mM MgCl_2_, 300 mM NaCl, 1 mM β-mercaptoethanol, 1% Triton-X, 5 mM Imidazole, 10% Glycerol and protease inhibitor cocktail (Roche)). Lysates were incubated for 30 min on ice before sonication (5 μm amplitude for 6 × 5 s), and clarification at 25,000*g* for 20 min at 4 °C. Tiam1-His protein was then purified using Ni^2+^-NTA agarose beads (Qiagen) according to the supplier’s protocol. Protein was eluted with Buffer A containing 100 mM Imidazole, and subsequently subject to desalting and buffer exchange using a PD-10 column (GE Healthcare) to a storage buffer (50 mM Tris-HCl, pH 7.5, 150 mM NaCl, 5 mM MgCl_2_, 1 mM β-mercaptoethanol and 10% Glycerol). Protein was stored at −80 °C.

### Kinase assays

For *in vitro* kinase assays, GST-tagged kinases were obtained from ProQinase (Freibury, Germany). Purified Tiam1-His (∼50 ng) or beads with immunoprecipitated Tiam1-HA were incubated with ∼100 ng kinase in kinase assay buffer (70 mM HEPES, 3 mM MgCl_2_, 3 mM MnCl_2_, 3 μM Na-orthovanadate, 1.2 mM DTT, 50 μg ml^−1^ PEG_20,000_ and 1-2 mM ATP) with shaking at 30 °C for 40 min. The reaction was stopped by addition of SDS–PAGE sample buffer (NuPAGE, Invitrogen) and analysed by SDS–PAGE.

### Halo pulse-chase assay

Halo pulse-chase assay was carried out as previously described^39^. In brief, dox-treated MDCK II +Tiam1-Halo were treated with 50 nM HaloTag TMR ligands (Promega) for 10 min, washed three times in PBS, and media containing 50 μM of the blocking reagent succinimidyl ester (O4) (Promega) was added to block labelling of newly synthesized protein. Cells were lysed at appropriate time points and analysed by immunoblotting with HaloTag antibody (Promega).

### Centrosome preparation

Centrosomes were prepared essentially as described previously^59^ with some modifications. In brief, control or MDCK II cells inducibly expressing Tiam1 shRNA were treated with 5 μM Cytochalasin D (Sigma) and 10 μM Nocodazole (Sigma) in DMEM for 1 h before harvesting. All subsequent steps were performed on ice or at 4 °C. Cells were washed successively in PBS, 0.1x PBS/8% sucrose and finally 8% sucrose, before being lysed (1 mM Tris-pH 8.0, 0.5% triton, 0.1% β-mercaptoethanol, 0.5 mM MgCl_2_ and EDTA-free Complete protease inhibitor cocktail (Roche)). Lysates were cleared by 5 min centrifugation at 1,500*g* and passing through a 33-μm filter, before supplementing with PIPES and EDTA to final concentrations of 10 and 1 mM, respectively. The lysates were layered over a 60% sucrose (w/v) cushion (in PE (10 mM PIPES-pH 7.0, 1 mM EDTA) containing 0.1% triton) and centrifuged at 25,000*g* for 30 min to sediment centrosomes onto the cushion. The upper fraction was removed, while the remaining lower fraction and sucrose cushion, enriched for centrosomes, were thoroughly mixed (resulting in ∼30% sucrose (w/v) solution) and loaded onto a discontinuous sucrose gradient (70, 50 and 40% (w/v) in PE containing 0.1% triton), and centrifuged at 100,000*g* for 1 h. Fractions (400 μl) were collected from the bottom; each fraction was diluted with 1 ml of PE buffer and centrosomes were recovered by centrifugation at 16,000*g* for 20 min. Supernatants were removed and the pelleted centrosomes re-suspended in SDS sample buffer and processed for SDS–PAGE as described above.

### Flow cytometry for cell cycle analysis

Cells were collected by trypsinization and fixed in 70% ethanol/PBS for at least 16 h at −20 °C. Cells were permeabilized and blocked in a solution of 0.1% triton/0.5% BSA, stained with MPM2 antibody (to label mitotic cells), then anti-mouse APC secondary antibody, and re-suspended in a solution containing 50 μg ml^−1^ propidium iodide and 100 μg ml^−1^ RNAse. Cells were analysed for PI and APC fluorescence on a LSRII analyser (BD Biosciences) using FACSDiva software (version 6.1.1) (BD Biosciences). Cell cycle stage was quantified using FlowJo (Tree Star).

### Immunofluorescence

For immunofluorescence, cells were grown on coverslips and fixed with either 100% ice-cold methanol for 5 min at −20 °C (for Tiam1, P*S1466, γ-tubulin, GFP, centrin) or 3.7% formaldehyde for 20 min at room temperature (for HA, P*Aurora, P*Pak, γ-tubulin and α-tubulin). Staining was performed as described previously^36^ by permeabilization in 0.5% triton in PBS and blocking in 1% BSA in PBS before successive incubation with primary and then secondary antibodies. Coverslips were mounted to slides using Prolong Antifade with DAPI (Molecular Probes).

### Microscopy and image analysis

For scoring chromosome congression errors, intercentrosomal distance, intensity of P*Pak and P*Aurora A on the centrosomes, and mitotic stage following monastrol treatment, images were recorded with a Deltavision Core (Applied Precision Instruments) system based around an Olympus IX71 microscope with illumination achieved by white light LED and a 300 W Xenon light source for fluorescence. The Sedat filter (Chroma, 89000) set was utilized for fluorescence imaging using a UPLSAPO 60XO 1.35NA objective, and image capture was via a Roper Cascade II 512B EMCCD camera and SoftWorx software (Applied Precision Instruments). Inter-centrosomal distance for prophase (cells with condensed chromosomes but intact nuclear envelope) and prometaphase (post-NEBD cells without an aligned metaphase plate) cells was measured in 3D using SoftWorx. For rescue experiments, only cells with positive HA or GFP staining were measured, except cells with very high expression that were excluded. Scoring of monopolar/bipolar spindles was also performed in SoftWorx; a separation of ⩾4 μm was considered bipolar. Scoring of chromosome congression errors was as described previously^7^. Centrosomal signal intensity (P*Pak and P*Aurora A) was quantified in Image J by producing intensity plots for a rectangular section around the centrosomal area. For P*S1466 and Tiam1 (Fig. 2g,h and Supplementary Fig. 2b,d,e), images were acquired on a Leica TCS SP8 confocal microscope equipped with PMT and Hybrid (HyD) detectors, with the tunable white laser (WLL) for Alexa Fluor 488, Alexa Fluor 555 and Alexa Fluor 647, and 405 nm UV laser for DAPI. The × 100/1.4 NA oil immersion objective was used, and images were captured in LAS AF (3.0.1) leica software. For live imaging, cells in 96-well plates were imaged using an Opera Phenix (Perkin Elmer) with temperature and environmental controls (37 °C and 5% CO_2_), using a × 40 1.1 NA water immersion lens (Zeiss). Frames were captured at 5 min intervals. Movies were exported from the Columbus software and analysed manually in ImageJ; the first frame with evidence of chromosome condensation was taken as the start of prophase, and the first frame in which cells had clearly aligned chromosomes taken as the start of metaphase. Cells still in prophase at the end of the movie were censored (given value 0) while those reaching metaphase were given a value 1; data was then analysed in Prism using survival curve analysis. All images shown were analysed in Image J.

### Statistical analysis

Statistical differences between two sets of data were analysed in Microsoft Excel using a two-tailed paired or unpaired student’s *t*-test (test and *P* values are specified in figure legends). For live imaging experiments, data were analysed in Prism (GraphPad software) using the survival curve function; the Gehan-Breslow-Wilcoxon test was used to test for significance.

## Additional information

**How to cite this article:** Whalley, H. J. *et al*. Cdk1 phosphorylates the Rac activator Tiam1 to activate centrosomal Pak and promote mitotic spindle formation. *Nat. Commun.* 6:7437 doi: 10.1038/ncomms8437 (2015).

## Supplementary Material

Supplementary Information Supplementary Figures 1-7.

Supplementary Movie 1 Non-targeting control siRNA cell during live imaging in monastrol. MDCK II cells expressing histone-2B-GFP (H2B-GFP) and a-tubulin-RFP were transiently transfected with non-targeting siRNA (NT_Control) for 2 days then treated with 25 μm monastrol before being imaged using time-lapse confocal microscopy as described in Methods. Left panel: a-tubulin-RFP, middle panel: H2B-GFP, right panel: merge (α-tubulin-RFP= green, H2B-GFP=blue).

Supplementary Movie 2 Pak1#1 siRNA cell during live imaging in monastrol. MDCK II cells expressing histone-2B-GFP (H2B-GFP) and a-tubulin-RFP were transiently transfected with Pak1#1 siRNA for 2 days then treated with 25 μm monastrol before being imaged using timelapse confocal microscopy as described in Methods. Left panel: a-tubulin-RFP, middle panel: H2BGFP, right panel: merge (α-tubulin-RFP= green, H2B-GFP=blue)

Supplementary Movie 3 Pak2#1 siRNA cell during live imaging in monastrol. MDCK II cells expressing histone-2B-GFP (H2B-GFP) and a-tubulin-RFP were transiently transfected with Pak2#1 siRNA for 2 days then treated with 25 μm monastrol before being imaged using timelapse confocal microscopy as described in Methods. Left panel: a-tubulin-RFP, middle panel: H2BGFP, right panel: merge (α-tubulin-RFP= green, H2B-GFP=blue).

Supplementary Movie 4 Tiam1 siRNA cell during live imaging in monastrol. MDCK II cells expressing histone-2B-GFP (H2B-GFP) and a-tubulin-RFP were transiently transfected with Tiam1 siRNA for 2 days then treated with 25 μm monastrol before being imaged using time-lapse confocal microscopy as described in Methods. Left panel: a-tubulin-RFP, middle panel: H2B-GFP, right panel: merge (α-tubulin-RFP= green, H2B-GFP=blue).

## References

[b1] Blagden S. P. & Glover D. M. Polar expeditions - provisioning the centrosome for mitosis. Nat. Cell Biol. 5, 505–511 (2003). 12776127 10.1038/ncb0603-505

[b2] Fry A. M. . C-Nap1, a novel centrosomal coiled-coil protein and candidate substrate of the cell cycle–regulated protein kinase Nek2. J. Cell Biol. 141, 1563–1574 (1998). 9647649 10.1083/jcb.141.7.1563 PMC2133000

[b3] Tanenbaum M. E. & Medema R. H. Mechanisms of centrosome separation and bipolar spindle assembly. Dev. Cell 19, 797–806 (2010). 21145497 10.1016/j.devcel.2010.11.011

[b4] Ferenz N. P., Gable A. & Wadsworth P. Mitotic functions of kinesin-5. Semin. Cell Dev. Biol. 21, 255–259 (2010). 20109572 10.1016/j.semcdb.2010.01.019 PMC2844466

[b5] Tanenbaum M. E., Macurek L., Galjart N. & Medema R. H. Dynein, Lis1 and CLIP-170 counteract Eg5-dependent centrosome separation during bipolar spindle assembly. EMBO J. 27, 3235–3245 (2008). 19020519 10.1038/emboj.2008.242 PMC2609737

[b6] Whitehead C. M. & Rattner J. B. Expanding the role of HsEg5 within the mitotic and post-mitotic phases of the cell cycle. J. Cell Sci. 111, 2551–2561 (1998). 9701554 10.1242/jcs.111.17.2551

[b7] Woodcock S. A. . Tiam1-Rac signaling counteracts Eg5 during bipolar spindle assembly to facilitate chromosome congression. Curr. Biol. 20, 669–675 (2010). 20346677 10.1016/j.cub.2010.02.033 PMC2989435

[b8] DeBonis S. . In vitro screening for inhibitors of the human mitotic kinesin Eg5 with antimitotic and antitumor activities. Mol. Cancer Ther. 3, 1079–1090 (2004). 15367702

[b9] Mayer T. U. . Small molecule inhibitor of mitotic spindle bipolarity identified in a phenotype-based screen. Science 286, 971–974 (1999). 10542155 10.1126/science.286.5441.971

[b10] Wojcik E. J. . Kinesin-5: Cross-bridging mechanism to targeted clinical therapy. Gene 531, 133–149 (2013). 23954229 10.1016/j.gene.2013.08.004 PMC3801170

[b11] van Heesbeen RGHP, Tanenbaum M. E. & Medema R. H. Balanced activity of three mitotic motors is required for bipolar spindle assembly and chromosome segregation. Cell Rep. 8, 948–956 (2014). 25127142 10.1016/j.celrep.2014.07.015

[b12] Mountain V. . The kinesin-related protein, HSET, opposes the activity of Eg5 and cross-links microtubules in the mammalian mitotic spindle. J. Cell Biol. 147, 351–366 (1999). 10525540 10.1083/jcb.147.2.351 PMC2174226

[b13] Mertens A. E., Roovers R. C. & Collard J. G. Regulation of Tiam1-Rac signalling. FEBS Lett. 546, 11–16 (2003). 12829230 10.1016/s0014-5793(03)00435-6

[b14] Malliri A. . Mice deficient in the Rac activator Tiam1 are resistant to Ras-induced skin tumours. Nature 417, 867–871 (2002). 12075356 10.1038/nature00848

[b15] Nigg E. A. Mitotic kinases as regulators of cell division and its checkpoints. Nat. Rev. Mol. Cell Biol. 2, 21–32 (2001). 11413462 10.1038/35048096

[b16] Gavet O. & Pines J. Activation of cyclin B1–Cdk1 synchronizes events in the nucleus and the cytoplasm at mitosis. J. Cell Biol. 189, 247–259 (2010). 20404109 10.1083/jcb.200909144 PMC2856909

[b17] Clute P. & Pines J. Temporal and spatial control of cyclin B1 destruction in metaphase. Nat. Cell Biol. 1, 82–87 (1999). 10559878 10.1038/10049

[b18] Jackman M., Lindon C., Nigg E. A. & Pines J. Active cyclin B1-Cdk1 first appears on centrosomes in prophase. Nat. Cell Biol. 5, 143–148 (2003). 12524548 10.1038/ncb918

[b19] Lindqvist A., Rodríguez-Bravo V. & Medema R. H. The decision to enter mitosis: feedback and redundancy in the mitotic entry network. J. Cell Biol. 185, 193–202 (2009). 19364923 10.1083/jcb.200812045 PMC2700378

[b20] Bertran M. T. . Nek9 is a Plk1 activated kinase that controls early centrosome separation through Nek6/7 and Eg5. EMBO J. 30, 2634–2647 (2011). 21642957 10.1038/emboj.2011.179 PMC3155310

[b21] Blangy A. . Phosphorylation by p34cdc2 regulates spindle association of human Eg5, a kinesin-related motor essential for bipolar spindle formation in vivo. Cell 83, 1159–1169 (1995). 8548803 10.1016/0092-8674(95)90142-6

[b22] Smith E. . Differential control of Eg5-dependent centrosome separation by Plk1 and Cdk1. EMBO J. 30, 2233–2245 (2011). 21522128 10.1038/emboj.2011.120 PMC3117641

[b23] Kyung S. L., Jung-Eun P., Young Hwi K., Tae-Sung K. & Jeong K. B. Mechanisms underlying Plk1 Polo-Box Domain-mediated biological processes and their physiological significance. Mol. Cells 37, 286–294 (2014). 24722413 10.14348/molcells.2014.0002 PMC4012076

[b24] Mardin B. R., Agircan F. G., Lange C. & Schiebel E. Plk1 controls the Nek2A-PP1γ antagonism in centrosome disjunction. Curr. Biol. 21, 1145–1151 (2011). 21723128 10.1016/j.cub.2011.05.047

[b25] Barr A. R. & Gergely F. Aurora-A: the maker and breaker of spindle poles. J. Cell. Sci. 120, 2987–2996 (2007). 17715155 10.1242/jcs.013136

[b26] Manser E., Leung T., Salihuddin H., Zhao Z. S. & Lim L. A brain serine/threonine protein kinase activated by Cdc42 and Rac1. Nature 367, 40–46 (1994). 8107774 10.1038/367040a0

[b27] Radu M., Semenova G., Kosoff R. & Chernoff J. Pak signalling during the development and progression of cancer. Nat. Rev. Cancer 14, 13–25 (2014). 24505617 10.1038/nrc3645 PMC4115244

[b28] Hofmann C., Shepelev M. & Chernoff J. The genetics of Pak. J. Cell. Sci. 117, 4343–4354 (2004). 15331659 10.1242/jcs.01392

[b29] Li F. . p21-activated kinase 1 interacts with and phosphorylates histone H3 in breast cancer cells. EMBO Rep. 3, 767–773 (2002). 12151336 10.1093/embo-reports/kvf157 PMC1084211

[b30] May M., Schelle I., Brakebusch C., Rottner K. & Genth H. Rac1-dependent recruitment of Pak2 to G2 phase centrosomes and their roles in the regulation of mitotic entry. Cell Cycle 13, 2210–2220 (2014). 10.4161/cc.29279 PMC4111676 24840740

[b31] Maroto B., Ye M. B., von Lohneysen K., Schnelzer A. & Knaus U. G. P21-activated kinase is required for mitotic progression and regulates Plk1. Oncogene 27, 4900–4908 (2008). 18427546 10.1038/onc.2008.131

[b32] Ando Y., Yasuda S., Oceguera-Yanez F. & Narumiya S. Inactivation of Rho GTPases with Clostridium difficile Toxin B impairs centrosomal activation of Aurora-A in G2/M transition of HeLa cells. Mol. Biol. Cell 18, 3752–3763 (2007). 17634283 10.1091/mbc.E07-03-0281 PMC1995717

[b33] Faure S., Vigneron S., Doree M. & Morin N. A member of the Ste20/PAK family of protein kinases is involved in both arrest of Xenopus oocytes at G2/prophase of the first meiotic cell cycle and in prevention of apoptosis. EMBO J. 16, 5550–5561 (1997). 9312014 10.1093/emboj/16.18.5550 PMC1170187

[b34] Zhao Z. S., Lim J. P., Ng Y. W., Lim L. & Manser E. The GIT-associated kinase Pak targets to the centrosome and regulates Aurora-A. Mol. Cell 20, 237–249 (2005). 16246726 10.1016/j.molcel.2005.08.035

[b35] Fleming I. N., Elliott C. M., Buchanan F. G., Downes C. P. & Exton J. H. Ca^2+^/calmodulin-dependent protein kinase II regulates Tiam1 by reversible protein phosphorylation. J. Biol. Chem. 274, 12753–12758 (1999). 10212259 10.1074/jbc.274.18.12753

[b36] Woodcock S. A. . SRC-induced disassembly of adherens junctions requires localized phosphorylation and degradation of the Rac activator Tiam1. Mol. Cell 33, 639–653 (2009). 19285946 10.1016/j.molcel.2009.02.012

[b37] Miyamoto Y., Yamauchi J., Tanoue A., Wu C. & Mobley W. C. TrkB binds and tyrosine-phosphorylates Tiam1, leading to activation of Rac1 and induction of changes in cellular morphology. Proc. Natl Acad. Sci. USA 103, 10444–10449 (2006). 16801538 10.1073/pnas.0603914103 PMC1502477

[b38] Nigg E. A. Cellular substrates of p34cdc2 and its companion cyclin-dependent kinases. Trends Cell Biol. 3, 296–301 (1993). 14731846 10.1016/0962-8924(93)90011-o

[b39] Yamaguchi K., Inoue S., Ohara O. & Nagase T. Pulse-chase experiment for the analysis of protein stability in cultured mammalian cells by covalent fluorescent labeling of fusion proteins. Methods Mol. Biol. 577, 121–131 (2009). 19718513 10.1007/978-1-60761-232-2_10

[b40] Licciulli S. . FRAX597, a small molecule inhibitor of the p21-activated kinases, inhibits tumorigenesis of Neurofibromatosis Type 2 (NF2)-associated schwannomas. J. Biol. Chem. 288, 29105–29114 (2013). 23960073 10.1074/jbc.M113.510933 PMC3790009

[b41] Girdler F. . Validating Aurora B as an anti-cancer drug target. J. Cell Sci. 119, 3664–3675 (2006). 16912073 10.1242/jcs.03145

[b42] Marumoto T. . Aurora-A Kinase maintains the fidelity of early and late mitotic events in HeLa cells. J. Biol. Chem. 278, 51786–51795 (2003). 14523000 10.1074/jbc.M306275200

[b43] Joukov V., Walter Johannes C. & De Nicolo A. The Cep192-organized Aurora A-Plk1 cascade is essential for centrosome cycle and bipolar spindle assembly. Mol. Cell 55, 578–591 (2014). 25042804 10.1016/j.molcel.2014.06.016 PMC4245277

[b44] Buchsbaum R. J., Connolly B. A. & Feig L. A. Regulation of p70 S6 kinase by complex formation between the Rac Guanine Nucleotide Exchange Factor (Rac-GEF) Tiam1 and the scaffold Spinophilin. J. Biol. Chem. 278, 18833–18841 (2003). 12531897 10.1074/jbc.M207876200

[b45] Buchsbaum R. J., Connolly B. A. & Feig L. A. Interaction of Rac exchange factors Tiam1 and Ras-GRF1 with a scaffold for the p38 mitogen-activated protein kinase cascade. Mol. Cell Biol. 22, 4073–4085 (2002). 12024021 10.1128/MCB.22.12.4073-4085.2002 PMC133864

[b46] Kollu S., Bakhoum S. F. & Compton D. A. Interplay of microtubule dynamics and sliding during bipolar spindle formation in mammalian cells. Curr. Biol. 19, 2108–2113 (2009). 19931454 10.1016/j.cub.2009.10.056 PMC2805786

[b47] Pakala S. B., Nair V. S., Reddy S. D. & Kumar R. Signaling-dependent phosphorylation of mitotic centromere-associated kinesin regulates microtubule depolymerization and its centrosomal localization. J. Biol. Chem. 287, 40560–40569 (2012). 23055517 10.1074/jbc.M112.399576 PMC3504770

[b48] Vadlamudi R. K. . p21-activated kinase 1 regulates microtubule dynamics by phosphorylating tubulin cofactor B. Mol. Cell Biol. 25, 3726–3736 (2005). 15831477 10.1128/MCB.25.9.3726-3736.2005 PMC1084301

[b49] Wittmann T., Bokoch G. M. & Waterman-Storer C. M. Regulation of microtubule destabilizing activity of Op18/Stathmin downstream of Rac1. J. Biol. Chem. 279, 6196–6203 (2004). 14645234 10.1074/jbc.M307261200

[b50] Zenke F. T. . p21-activated Kinase 1 phosphorylates and regulates 14-3-3 binding to GEF-H1, a microtubule-localized Rho exchange factor. J. Biol. Chem. 279, 18392–18400 (2004). 14970201 10.1074/jbc.M400084200

[b51] Chow H. Y. . p21-Activated Kinase 1 is required for efficient tumor formation and progression in a Ras-mediated skin cancer model. Cancer Res. 72, 5966–5975 (2012). 22983922 10.1158/0008-5472.CAN-12-2246 PMC3500416

[b52] Stebel A., Brachetti C., Kunkel M., Schmidt M. & Fritz G. Progression of breast tumors is accompanied by a decrease in expression of the Rho guanine exchange factor Tiam1. Oncol. Rep. 21, 217–222 (2009). 19082465

[b53] Hordijk P. L. . Inhibition of invasion of epithelial cells by Tiam1-Rac signaling. Science 278, 1464–1466 (1997). 9367959 10.1126/science.278.5342.1464

[b54] Ong C. C. . P21-activated kinase 1 (Pak1) as a therapeutic target in BRAF wild-type melanoma. J. Natl Cancer Inst. 105, 606–607 (2013). 23535073 10.1093/jnci/djt054

[b55] Michiels F. . Regulated membrane localization of Tiam1, mediated by the NH2-terminal pleckstrin homology domain, is required for Rac-dependent membrane ruffling and C-Jun NH2-terminal kinase activation. J. Cell Biol. 137, 387–398 (1997). 9128250 10.1083/jcb.137.2.387 PMC2139766

[b56] Mack N. A. . β2-syntrophin and Par-3 promote an apicobasal Rac activity gradient at cell–cell junctions by differentially regulating Tiam1 activity. Nat. Cell Biol. 14, 1169–1180 (2012). 23103911 10.1038/ncb2608 PMC3498067

[b57] Woodcock S. A., Jones R. C., Edmondson R. D. & Malliri A. A modified tandem affinity purification technique identifies that 14-3-3 proteins interact with Tiam1, an interaction which controls Tiam1 stability. J. Proteome Res. 12, 5629–5641 (2009). 10.1021/pr900716e 19899799

[b58] Unwin R. D. . Multiple reaction monitoring to identify sites of protein phosphorylation with high sensitivity. Mol. Cell. Proteomics 4, 1134–1144 (2005). 15923565 10.1074/mcp.M500113-MCP200

[b59] Bornens M., Paintrand M., Berges J., Marty M. C. & Karsenti E. Structural and chemical characterization of isolated centrosomes. Cell Motil. Cytoskeleton 8, 238–249 (1987). 3690689 10.1002/cm.970080305

