# Supplementary material for: Cdk1 phosphorylates the Rac activator Tiam1 to activate centrosomal Pak and promote mitotic spindle formation
Source: Nat Commun. 2015 Jun 16;6:7437. doi: 10.1038/ncomms8437 (PMC4490568; doi:10.1038/ncomms8437)

## SUPPLEMENTARY FIGURE LEGENDS

Supplementary Figure 1

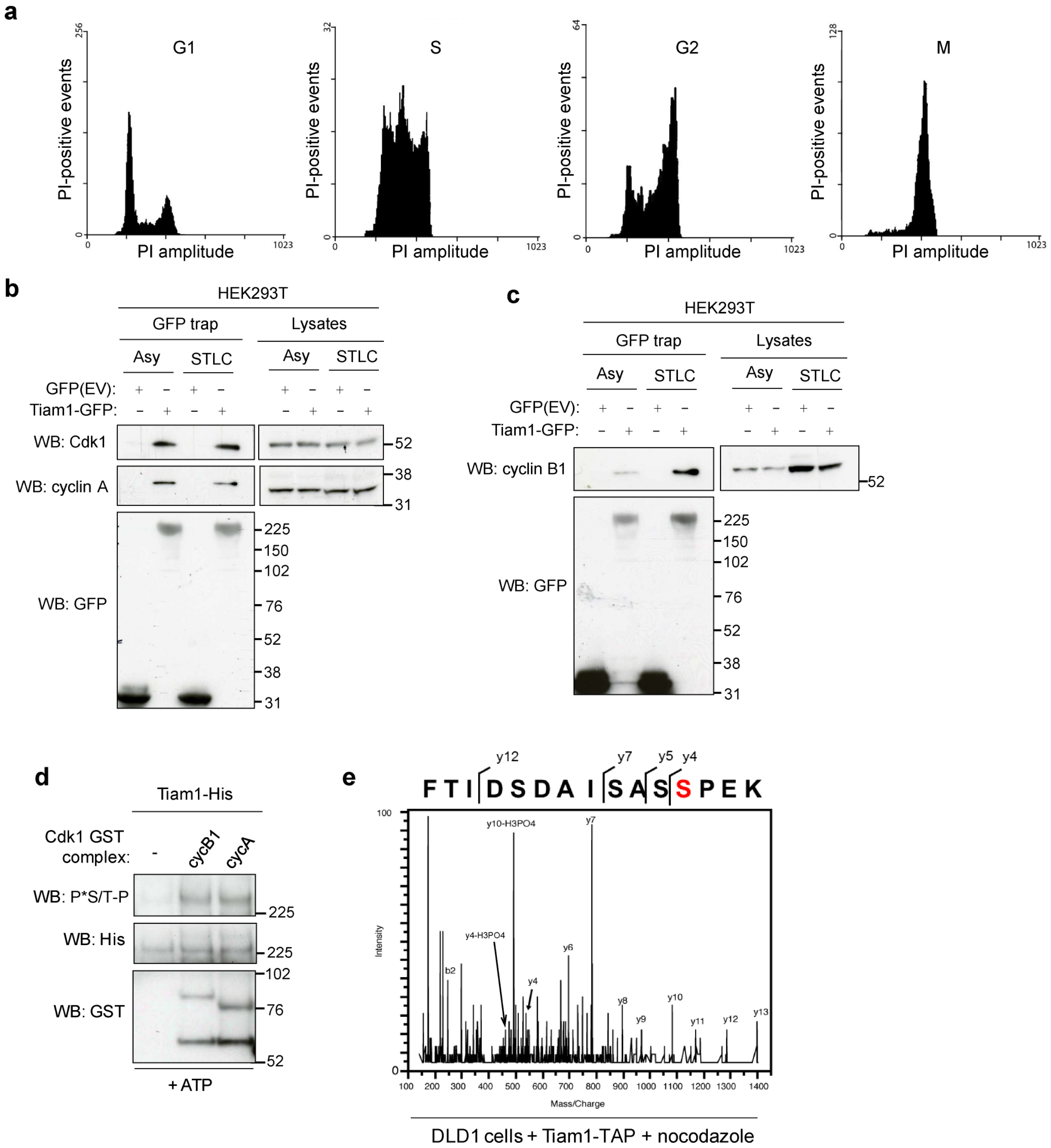


**Supplementary Figure 1. Cdk1-cyclin complexes interact with and phosphorylate Tiam1. (a)** MDCK II cells were synchronised in the indicated cell cycle stages as described in Methods and fixed. Cell cycle stage was assessed by FACS analysis following staining with propidium iodide (PI). **(b-c)** Lysates of asynchronous (Asy) or mitotically arrested (STLC) HEK293T cells expressing GFP only [GFP(EV)] or Tiam1-GFP were subjected to GFP-trap and analysed by immunoblotting for **(b)** Cdk1 and cyclin A or **(c)** cyclin B1 interactions. **(d)** Tiam1-His purified from insect cells was subjected to *in vitro* kinase assay with GST-tagged Cdk1-cyclin B1 or Cdk1-cyclin A complex as indicated. Samples were analysed by SDS-PAGE and phosphorylation measured using anti-P*-Thr-Pro antibody (P*S/T-P). **(e)** DLD1 cells stably expressing TAP-tagged Tiam1 (mouse) were arrested in mitosis using nocodazole, TAP performed, then Tiam1-TAP was isolated by SDS-PAGE and coomasie staining and the resulting band phospho-mapped by mass spectrometry. Shown is the product ion spectrum demonstrating phosphorylation of Tiam1 at S1466 with annotated peptide sequence and pertinent ions labelled. The series of y ions including y4-y7 & y12 confirm the peptide sequence with y4 & y4-$H_3PO_4$ further confirming the site of phosphorylation as S1466.

# Supplementary Figure 2

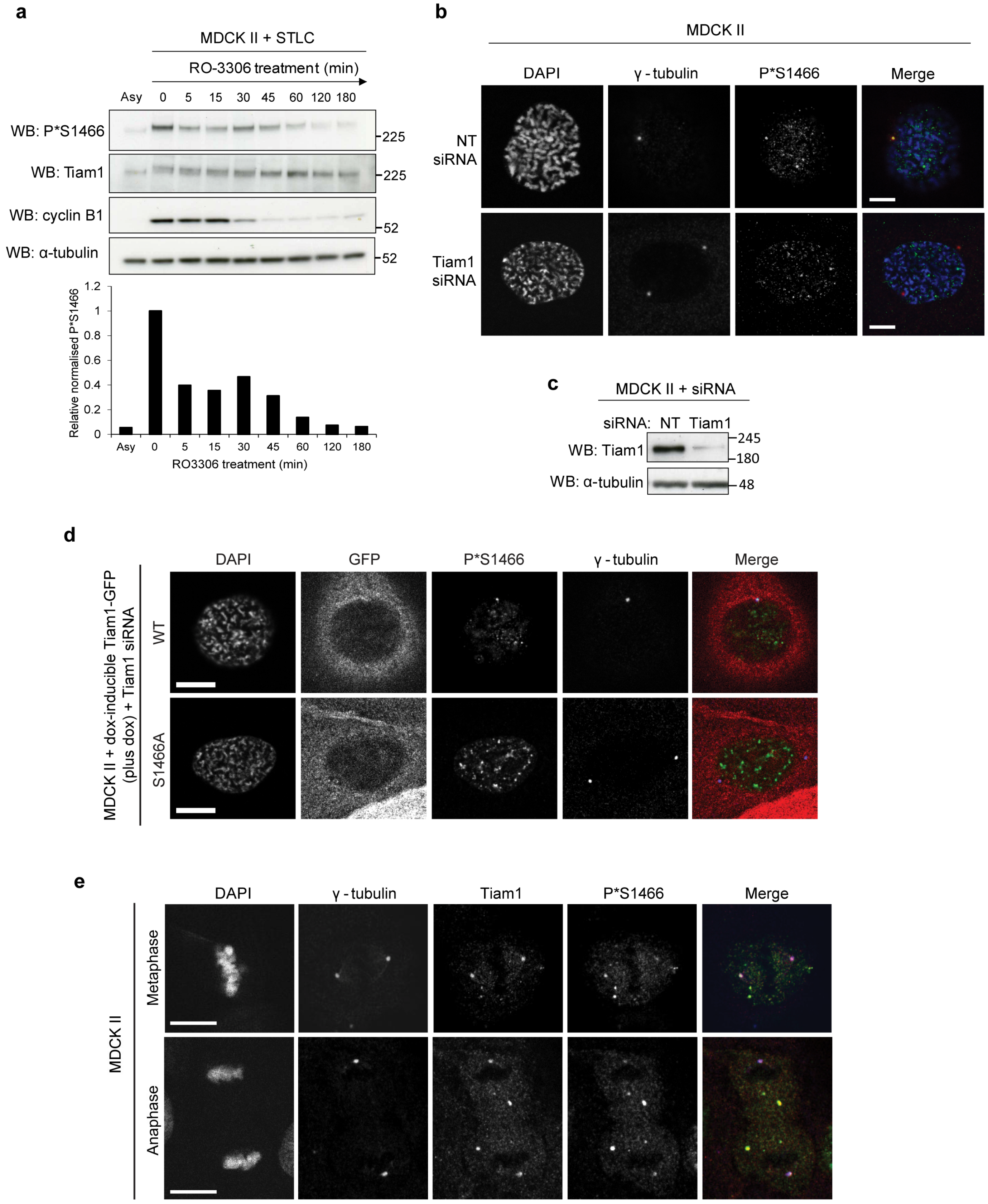

**Supplementary Figure 2. S1466 phosphorylation depends on Cdk1 and occurs on mitotic centrosomes.** **(a)** MDCK II cells were left asynchronous (Asy) or arrested in mitosis by STLC then treated with the Cdk1 inhibitor RO-3306 for the indicated times before lysis and analysis by immunoblotting with the indicated antibodies. Graph shows quantification of the band intensity for P*S1466 normalised to total Tiam1 over the time course, with the STLC-only sample (0 min) set as 1. **(b)** MDCK II cells were transfected with siRNA for Tiam1 or a non-targeting control (NT) as indicated, fixed and stained for immunofluorescence (IF) using antibodies against P*S1466(Tiam1) (green), γ-tubulin (red) and DAPI (blue). Representative images of prophase cells are shown. Scale bars: 10 µm. **(c)** MDCK II cells were transfected with siRNAs as in **(b)** and cells lysed and analysed by immunoblotting with the indicated antibodies. **(d)** MDCK II cells with dox-inducible over-expression of Tiam1-GFP (WT or S1466A as indicated) were transfected with siRNA and dox added 1 day later for 1 day. Cells were fixed and stained for IF using the indicated antibodies and DAPI. Representative prophase cells are shown. Merge:GFP = red, P*S1466 = green, γ-tubulin = blue. Scale bars: 10 µm. **(e)** MDCK II cells were fixed and stained by IF with the indicated antibodies and DAPI. Merge: Tiam1 = red, P*S1466 = green, γ-tubulin = blue. Representative metaphase and anaphase cells are shown. Scale bars: 10 µm.

# Supplementary Figure 3

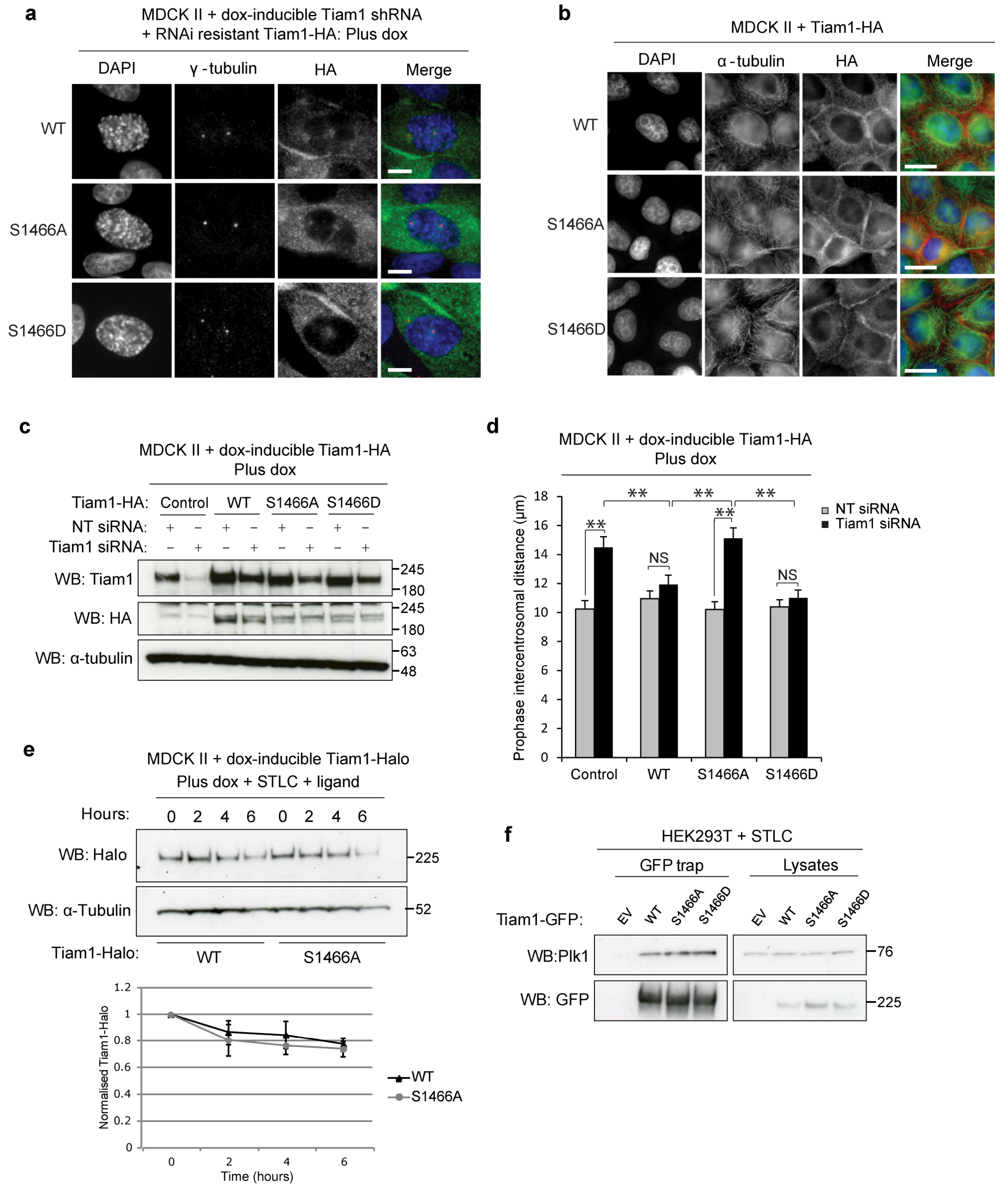

**Supplementary Figure 3. S1466 phosphorylation does not affect Tiam1 localisation, stability or its interaction with Plk1. (a)** MDCK II cells with dox-inducible Tiam1 shRNA and stable expression of the indicated HA-tagged Tiam1 constructs containing an RNAi-resistant mutation were treated with dox for 4 days to deplete endogenous Tiam1, fixed and stained for IF with antibodies against HA (green), γ-tubulin (red) and DAPI. Representative prophase cells are shown. Scale bars: 10 μm. **(b)** MDCK II cells with stable expression of the indicated HA-tagged Tiam1 constructs were fixed and stained for IF with antibodies against HA (red), α-tubulin (green) and DAPI. Representative cortical localisation is shown. Scale bars: 20 μm. **(c-d)** MDCK II cells expressing the indicated dox-inducible Tiam1-HA constructs were transfected with siRNAs for Tiam1 or a non-targeting control (NT) and dox added 1 day later for 1 day**. (c)** Cells were lysed and analysed by immunoblotting with the indicated antibodies. **(d)** Cells were fixed and stained by IF with antibodies against HA, γ-tubulin and DAPI, and intercentrosomal distance in prophase was measured in cells with positive HA signal (>60 cells over at least 3 independent experiments). Graph shows mean + s.e.m. Unpaired two-sided t-test: **$p<0.01$. **(e)** MDCK II cells stably expressing Tiam1-Halo (either WT or S1466A as indicated) arrested in mitosis with STLC treatment were labelled with Halo ligand as described in Methods and lysed at the indicated time points. Decrease in labelled protein was analysed by immunoblotting with Halo antibody. Graph shows mean normalised (to 0 hour) protein level for WT and S1466A mutant, n=4 (2 and 4 hour time points) or n=8 (6 hour time point), error bars show s.e.m. **(f)** HEK293T cells expressing the indicated GFP constructs were arrested in mitosis (STLC) and lysates subjected to GFP-trap followed by immunoblotting for Plk1.

## Supplementary Figure 4

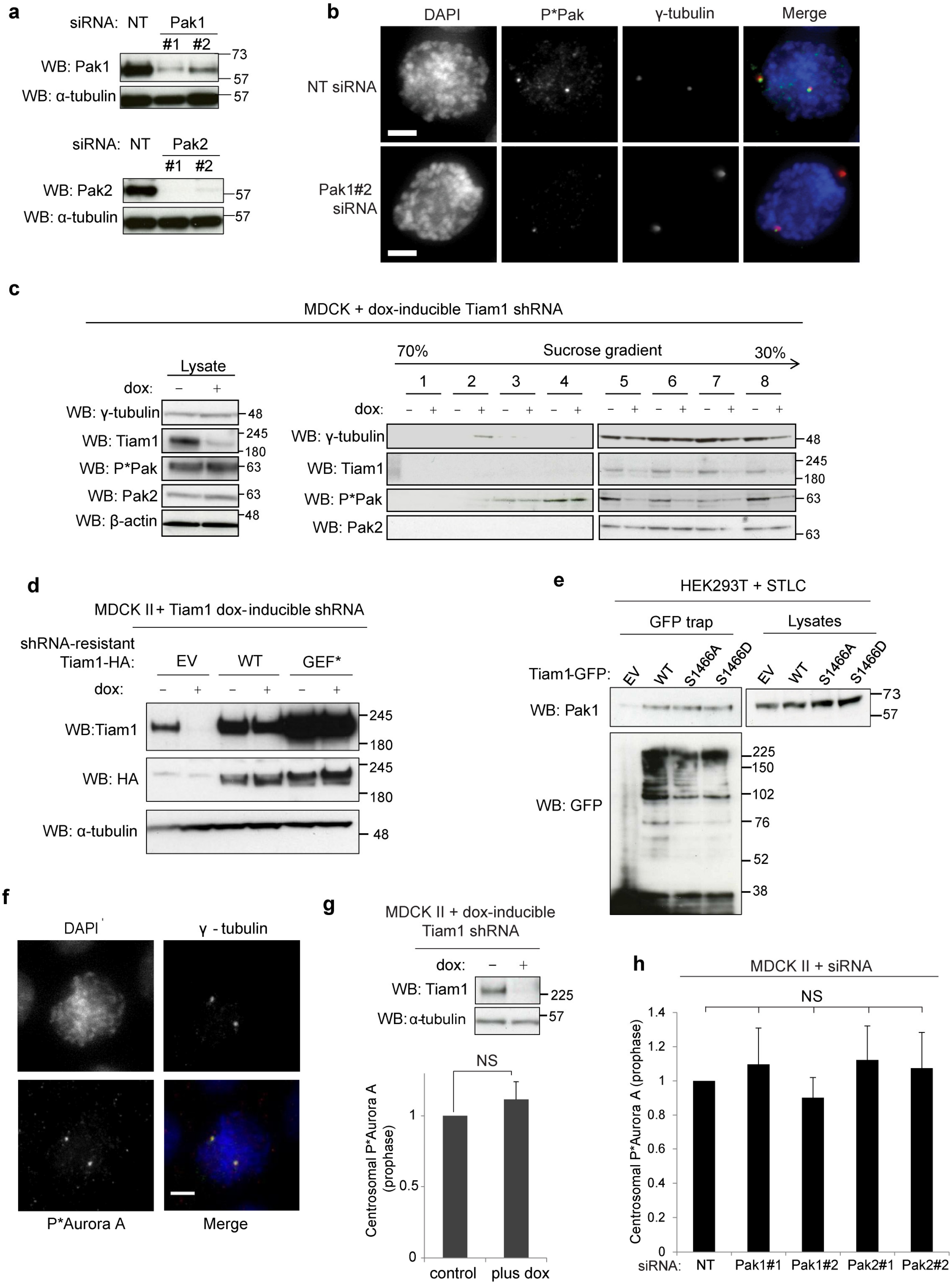

**Supplementary Figure 4. Tiam1 GEF activity is required to activate Pak1/2 but Tiam1-Rac-Pak1/2 signalling does not regulate centrosomal Aurora A activity. (a)** MDCK II cells were treated with siRNAs to Pak1/2 or a non-targeting control (NT) as indicated and 2 days later lysed and analysed for immunoblotting with the indicated antibodies. **(b)** MDCK II cells were treated with siRNAs to Pak1 or a non-targeting control (NT) as indicated, fixed and stained for IF with antibodies against auto-phosphorylation sites of Pak1/2 (P*Pak) (green), γ-tubulin (red) and DAPI. Representative prophase cells are shown. Scale bars: 5 μm. **(c)** MDCK II cells with dox-inducible Tiam1 shRNA were treated for 3 days with or without dox as indicated, lysed and subjected to centrosome prep by sucrose gradient ultracentrifugation as described in Methods. Following SDS-PAGE, protein levels in the initial cell lysate or the sucrose gradient fractions were analysed by immunoblotting with the indicated antibodies. **(d)** MDCK II cells expressing dox-inducible Tiam1 shRNA and the indicated RNAi-resistant Tiam1 constructs were treated for 4 days with or without dox as indicated, lysed and analysed by immunoblotting with the indicated antibodies. This blot relates to the data in Fig. 4d. **(e)** HEK293T cells expressing GFP only (EV) or Tiam1-GFP (WT or phospho-mutant forms as indicated) were arrested in mitosis by STLC treatment, then lysates subjected to GFP-trap. Following SDS-PAGE, interaction with Pak1 was analysed by immunoblotting. **(f)** MDCK II cells were fixed and stained for IF with antibodies against P*T288 of Aurora A (P*Aurora A - red), γ-tubulin (green) and DAPI (blue). Representative staining in control prophase cells is shown. Scale bar: 5 μm. **(g)** MDCK II cells with dox-inducible Tiam1 shRNA were treated for 4 days with (plus dox) or without dox (control). Cells were either lysed and analysed for Tiam1 level by immunoblotting, or stained as in **(f)** and centrosomal P*Aurora A level was quantified as described in Methods (n=3, >30 centrosomes per replicate). **(h)** MDCK II cells were treated with siRNAs to Pak1, Pak2 or a non-targeting control (NT) as indicated, fixed and stained as in **(f)**, and centrosomal P*Aurora A level was quantified as described in Methods (n=3, >40 centrosomes per replicate). Graphs show mean of plus dox or siRNA normalised to control (or NT) + s.e.m. Paired two-sided t-test: NS = not significant.

Supplementary Figure 5

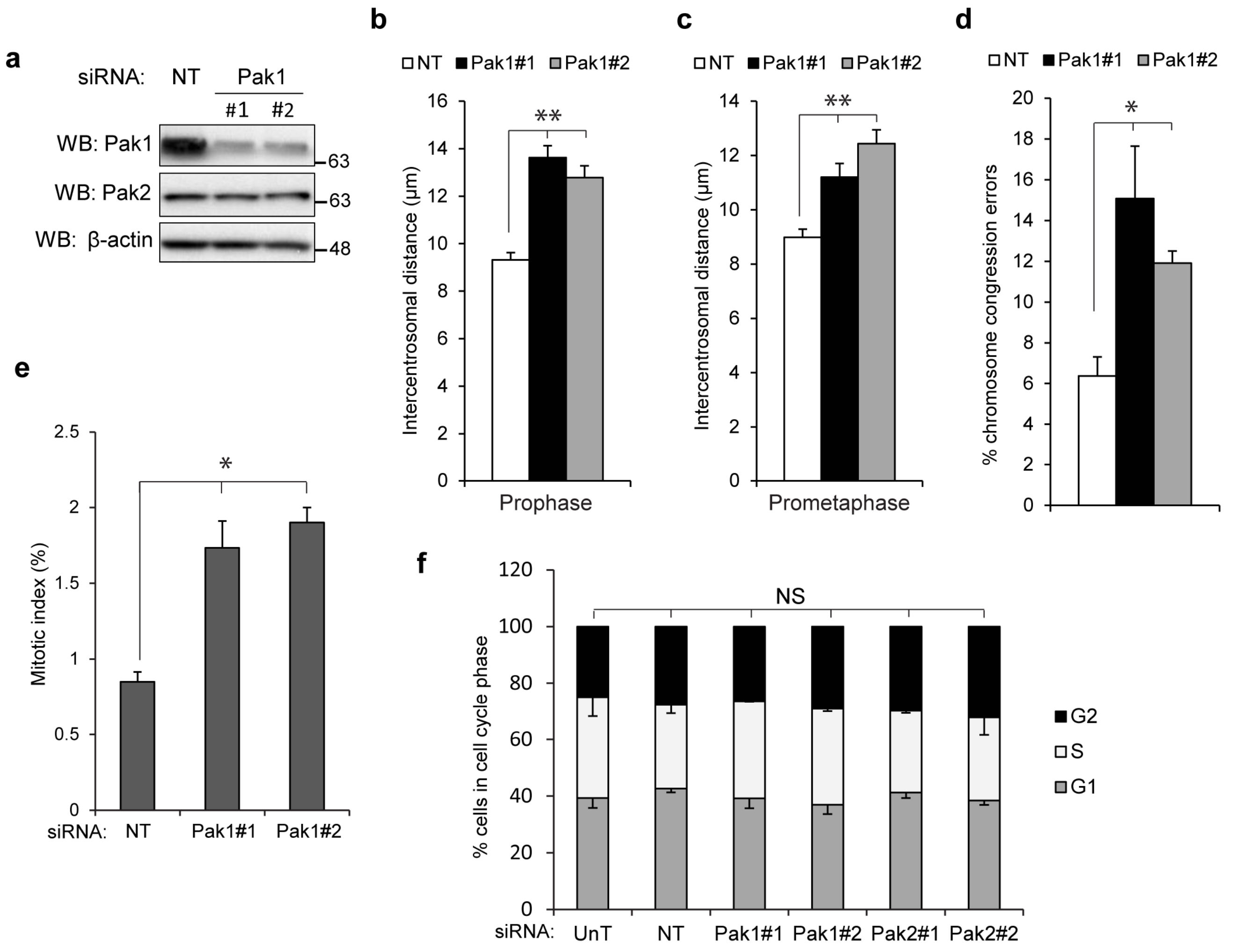


**Supplementary Figure 5. Pak1/2 knockdown antagonises centrosome separation and increases mitotic index.** **(a-d)** MDCK II cells were transiently transfected with two different siRNAs (#1, #2) against Pak1 or non-targeting control (NT) as indicated, then cells were either lysed and analysed by immunoblotting to confirm Pak1 knockdown **(a)** or fixed and stained for IF using antibodies against α-tubulin, γ-tubulin and DAPI. Intercentrosomal distance was measured for prophase **(b)** or prometaphase **(c)** cells (> 60 cells over at least 3 independent replicates). **(d)** Chromosome congression errors were scored (n=4). **(e-f)** MDCK II cells were transiently transfected with the indicated Pak siRNAs, then fixed and stained for FACS analysis with MPM2 antibody and propidium iodide (PI). **(e)** % MPM2 positive cells in the G2 peak represent mitotic index (n≥3). **(f)** Cell cycle phase was quantified from PI amplitude and each phase is represented as % of total cell cycle. All graphs show mean + s.e.m. Unpaired two-sided t-test: *p<0.05, **p<0.001.

# Supplementary Figure 6

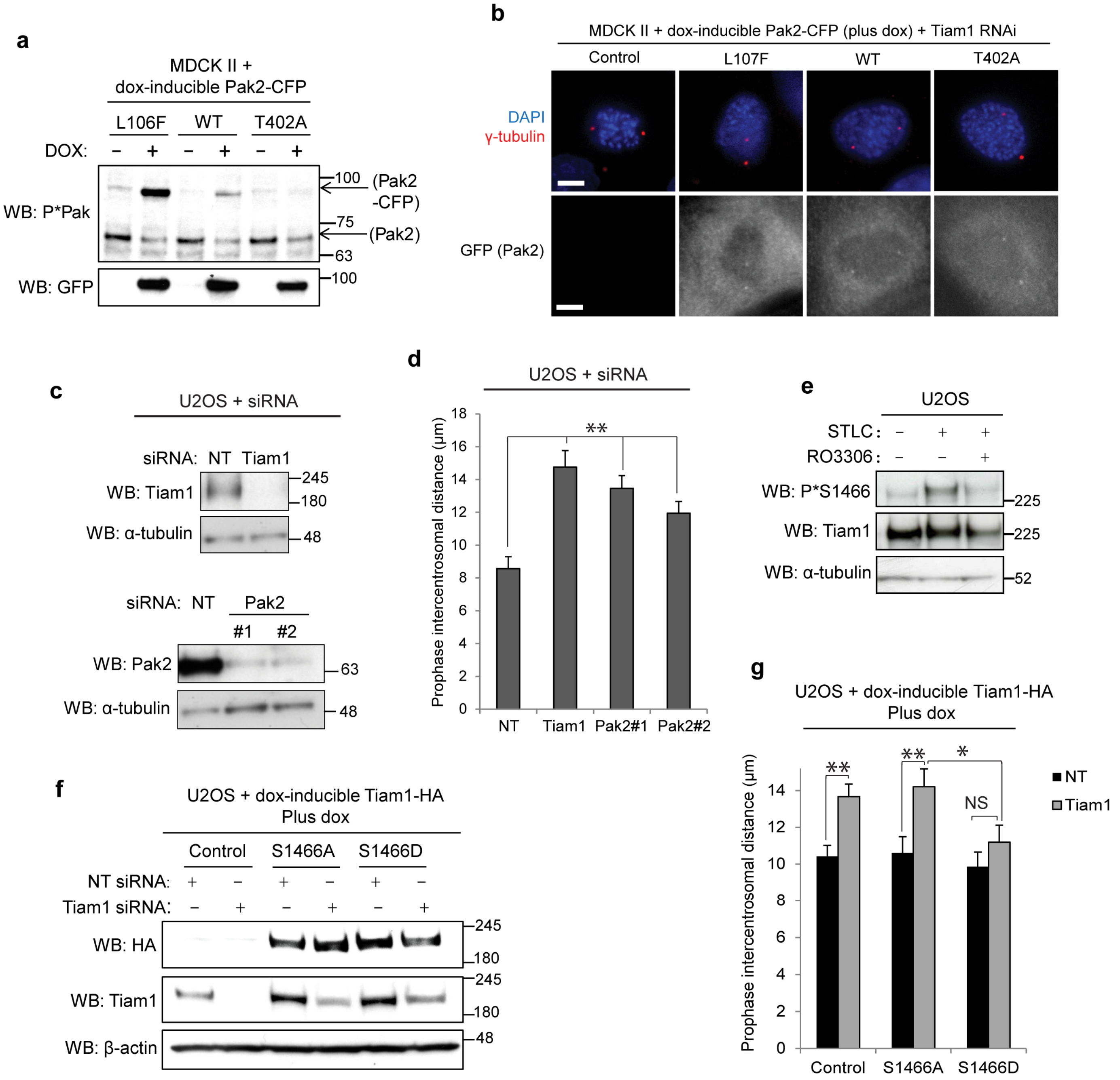

**Supplementary Figure 6. Pak activity is required for regulation of centrosome separation downstream of Tiam1 – analogous role for Tiam1-Rac-Pak signalling in U2OS cells. (a)** MDCK II cells with dox-inducible overexpression of Pak2-CFP: WT, L106F (kinase active), T402A (kinase inactive) were treated with or without dox for 1 day as indicated, lysed and analysed for Pak activity by immunoblotting with phospho-Pak1/2 antibody (P*Pak). **(b)** MDCK II cells with dox-inducible over-expression of WT, L106F (kinase active) or T402A (kinase inactive) Pak2-CFP or tet-ON construct only (Control) were transfected with Tiam1 siRNA and dox added 1 day later for 1 day. Cells were fixed and stained by IF with antibodies against γ-tubulin, GFP, and DAPI. Example IF images for prophase cells are shown (Merge: DAPI = blue, γ-tubulin = red). Scale bars:10 µm. **(c-d)** U2OS cells were transfected with the indicated siRNAs for Tiam1 or Pak2 or a non-targeting control (NT) and either lysed and analysed by immunoblotting with the indicated antibodies **(c)** or stained for IF using antibodies against α-tubulin, γ-tubulin and DAPI and intercentrosomal distance in prophase measured (3 independent experiments, >25 cells/ rep) **(d)**. **(e)** U2OS cells were treated for 16 hours with STLC to arrest cells in mitosis then for 2 hours with RO-3306 (Cdk1 inhibitor) before being lysed and analysed by immunoblotting with the indicated antibodies. **(f-g)** U2OS with dox-inducible expression of RNAi-resistant Tiam1-HA (S1466A or S1466D as indicated) were transfected with the indicated siRNAs (NT=non-targeting control) and treated with dox for 2 days. **(f)** Cells were lysed and analysed by immunoblotting with the indicated antibodies. **(g)** Cells were fixed and stained for IF with antibodies against HA, γ-tubulin and DAPI and intercentrosomal distance in prophase measured in cells positive for HA (n≥3, >55 cells/replicate). All graphs show mean + s.e.m. Unpaired two-sided t-test: *p<0.05, **p<0.01, NS = not significant.

Supplementary Figure 7

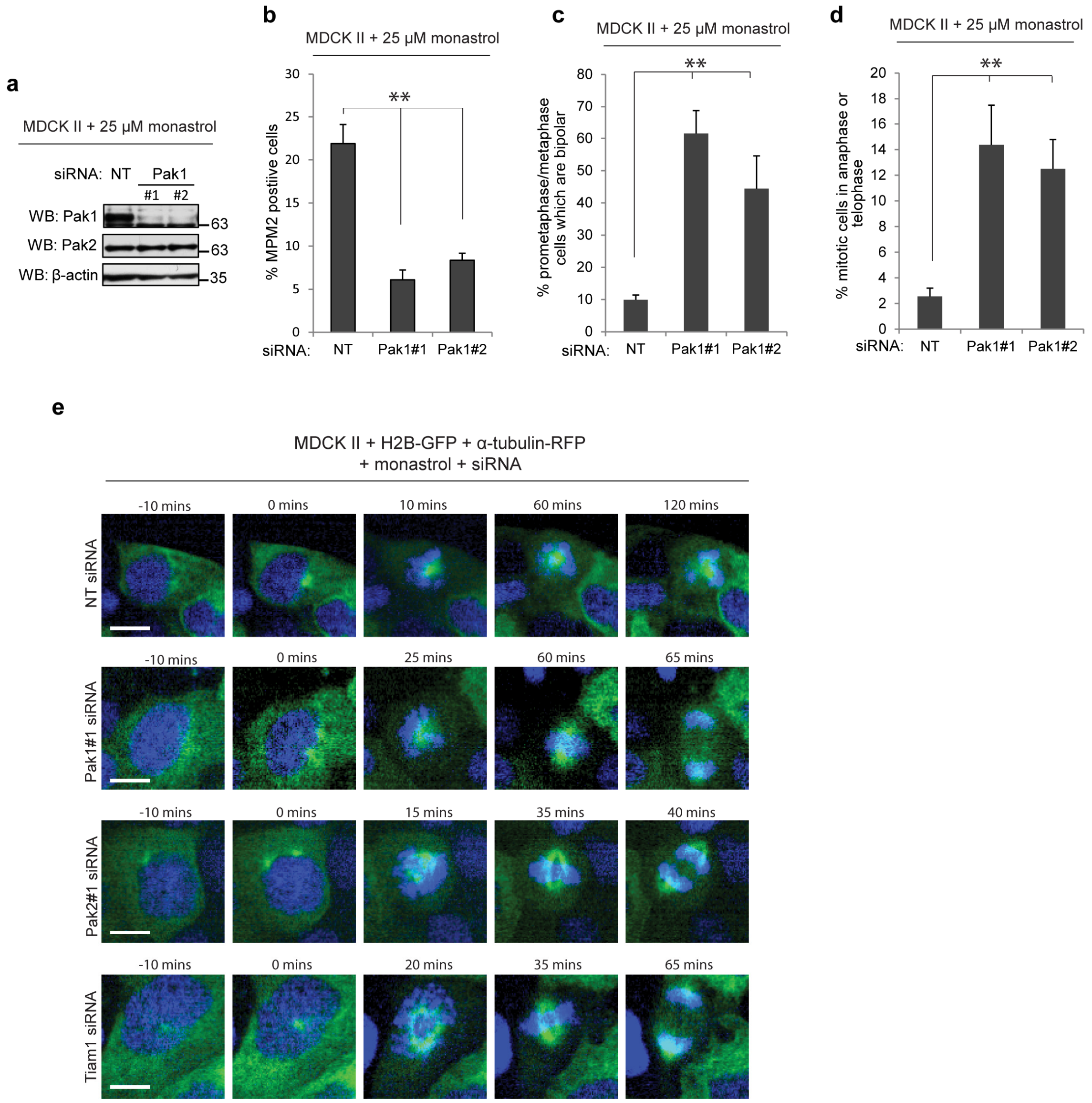


**Supplementary Figure 7. Tiam1 and Pak modulate responses to Eg5 inhibitors (a-d)** MDCK II cells were transiently transfected with two siRNAs against Pak1 or non-targeting control (NT) as indicated, and 2 days later treated with 25 µM monastrol for 16 hours. **(a)** Cells were lysed and analysed for knock-down by immunoblotting with the indicated antibodies. **(b)** Cells were fixed and stained for FACS analysis with propidium iodide and MPM2 antibody to detect % of mitotic cells (n ≥ 3, 10,000 cells/rep). **(c-d)** Cells were fixed and then stained by IF with antibodies against α-tubulin, γ-tubulin and DAPI. **(c)** Quantitation of the proportion of bipolar spindles (n ≥ 3, >100 cells/rep). **(d)** Quantitation of the proportion of mitotic cells in anaphase and telophase (n ≥ 3, >100 cells/rep). **(b-d)** All graphs show mean + s.e.m., unpaired two-sided t-test: **p<0.01. **(e)** MDCK II cells expressing histone-2B-GFP (H2B-GFP) and α-tubulin-RFP were transfected with the indicated siRNAs for 2 days then treated with 25 µm monastrol before being imaged using time-lapse confocal microscopy as described in Methods. Stills show progression of representative cells for each siRNA (these images relate to the data shown in Fig. 7f). Scale bars: 10 µm.

Full blot scans of immunoblot slices in main figures

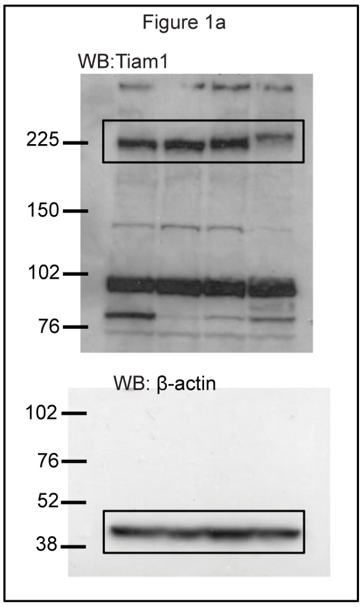


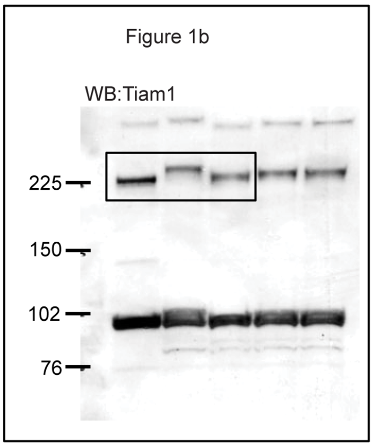


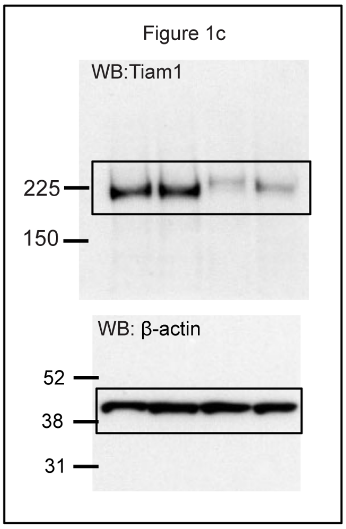


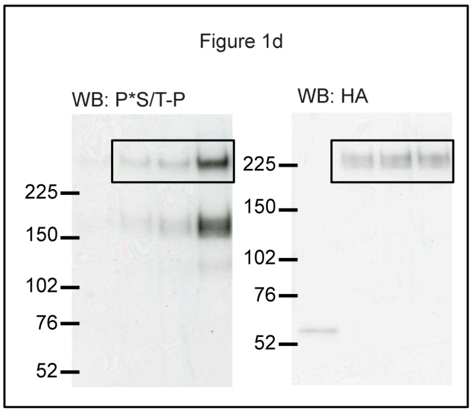


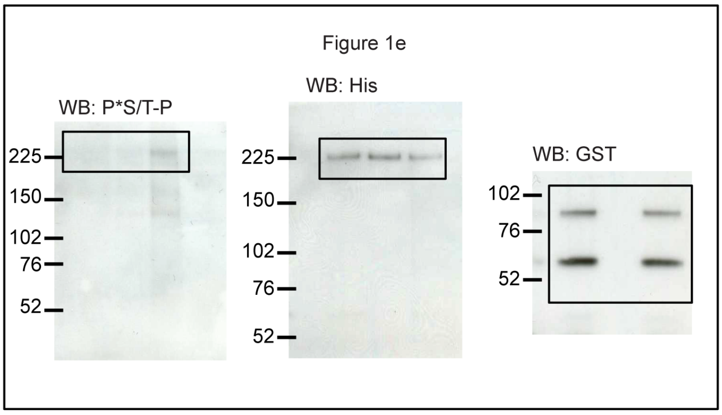


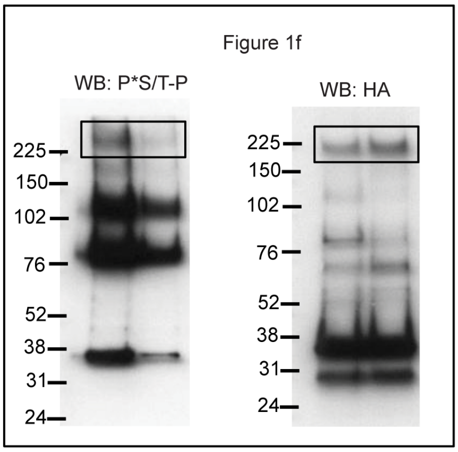

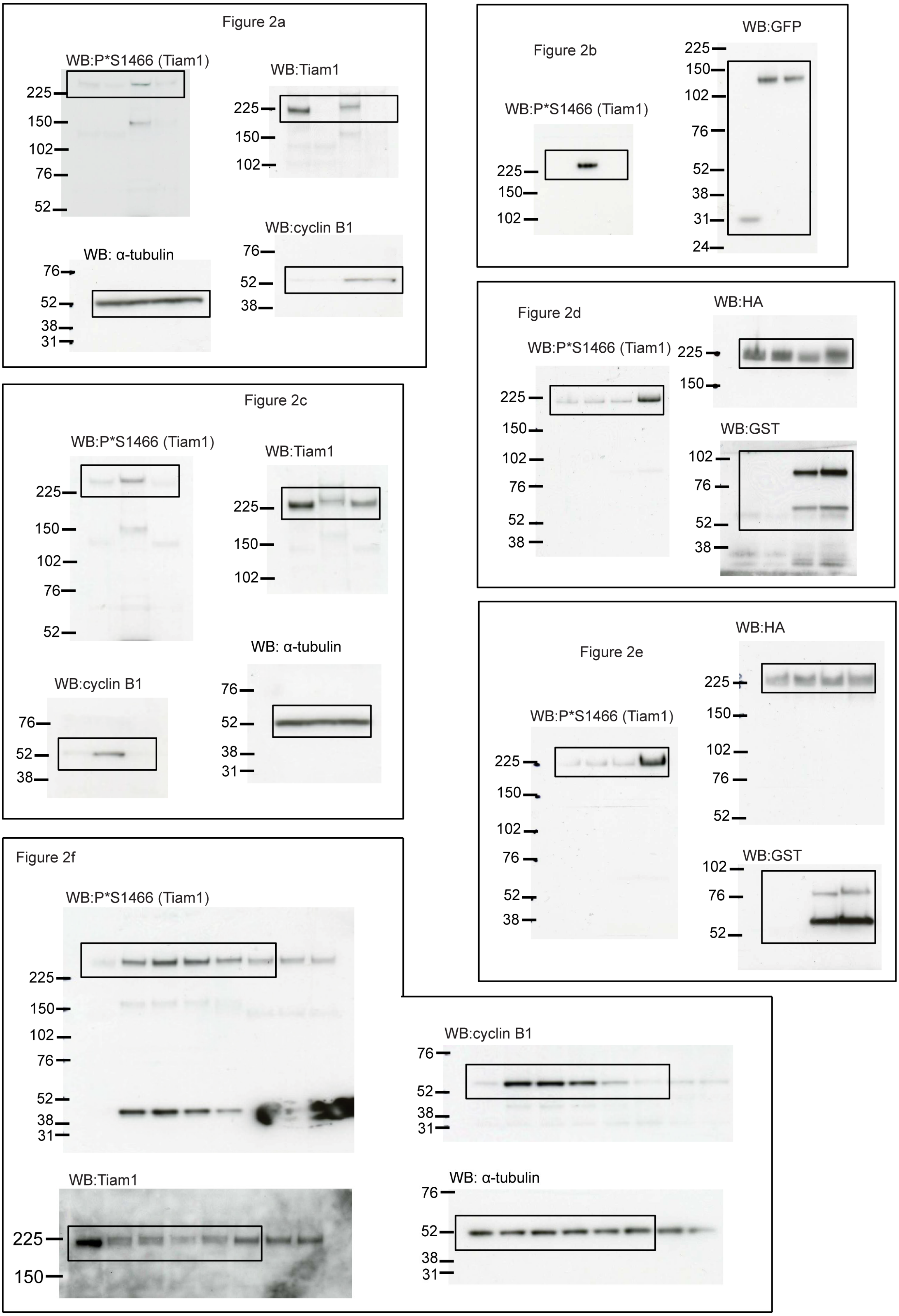

Figure 2a
WB:P*S1466 (Tiam1)
225
150
102
76
52
WB:Tiam1
225
150
102
WB: α-tubulin
76
52
38
31
WB:cyclin B1
76
52
38
Figure 2b
WB:P*S1466 (Tiam1)
225
150
102
WB:GFP
225
150
102
76
52
38
31
24
Figure 2c
WB:P*S1466 (Tiam1)
225
150
102
76
52
WB:Tiam1
225
150
102
WB:cyclin B1
76
52
38
WB: α-tubulin
76
52
38
31
Figure 2d
WB:P*S1466 (Tiam1)
225
150
102
76
52
38
WB:HA
225
150
WB:GST
102
76
52
38
Figure 2e
WB:P*S1466 (Tiam1)
225
150
102
76
52
38
WB:HA
225
150
102
76
52
WB:GST
102
76
52
Figure 2f
WB:P*S1466 (Tiam1)
225
150
102
76
52
38
31
WB:Tiam1
225
150
WB:cyclin B1
76
52
38
31
WB: α-tubulin
76
52
38
31

Figure 3b

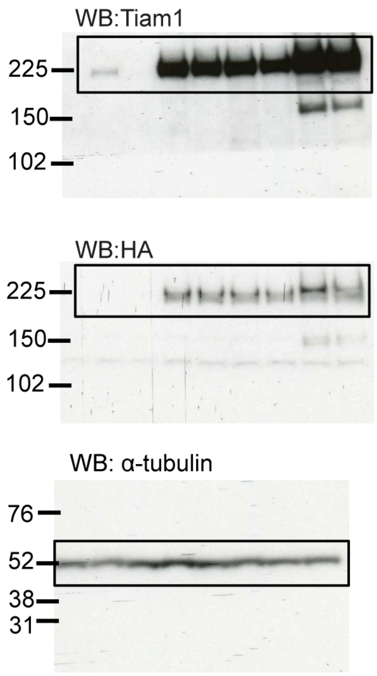


Figure 3h

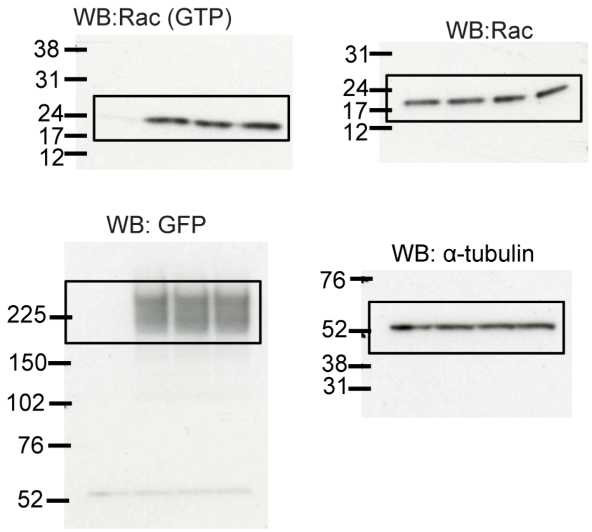


Figure 4f

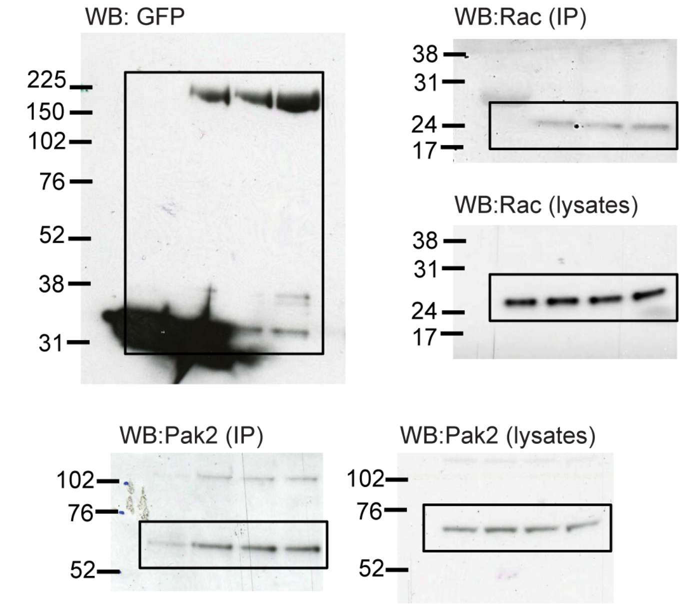


Figure 5a

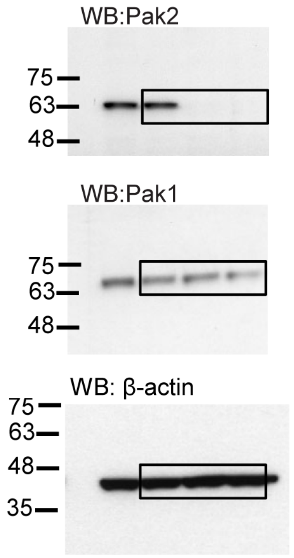


Figure 5f

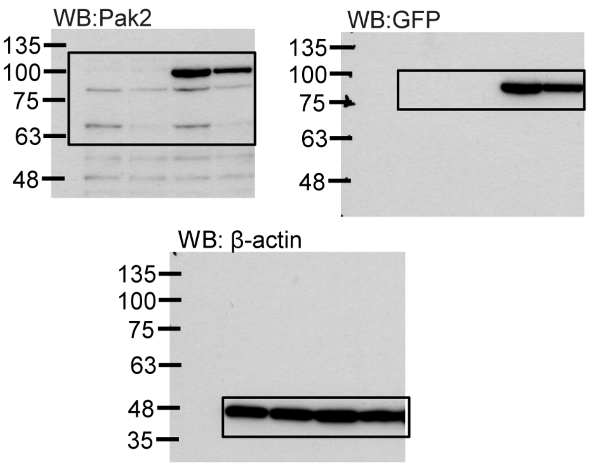

Figure 6a

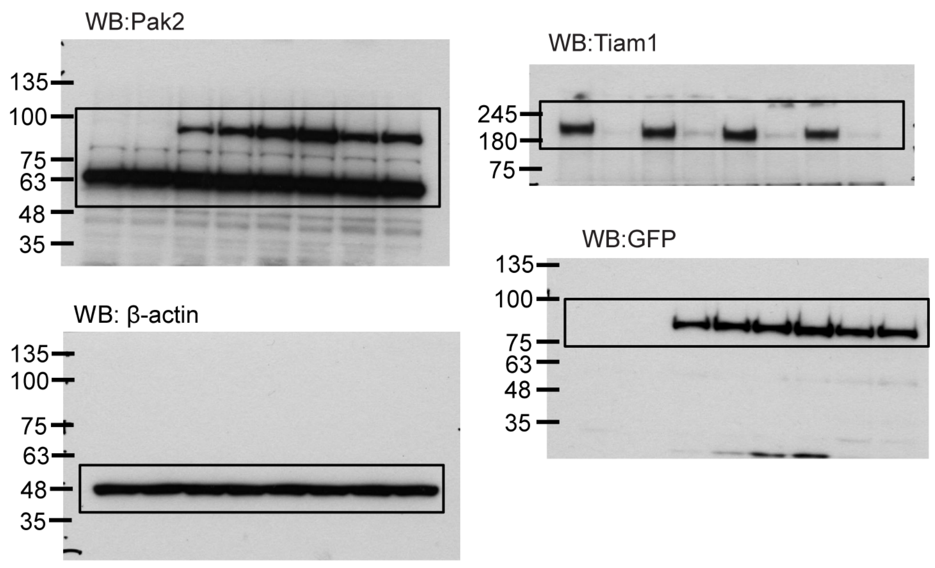


Figure 6c

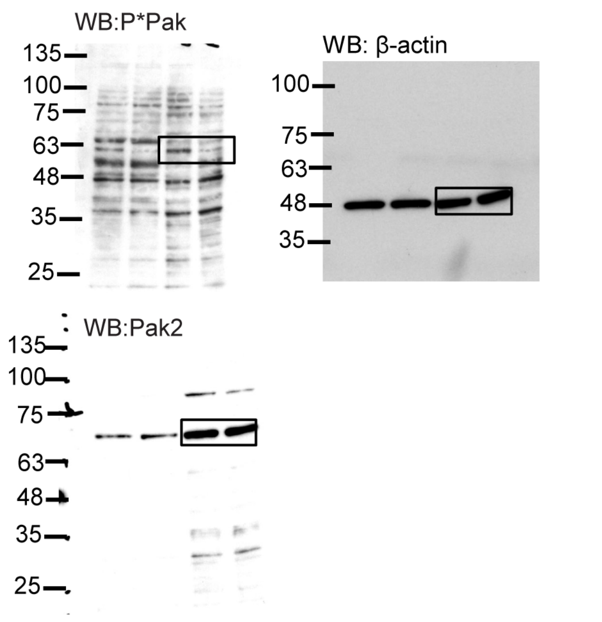


Figure 7a

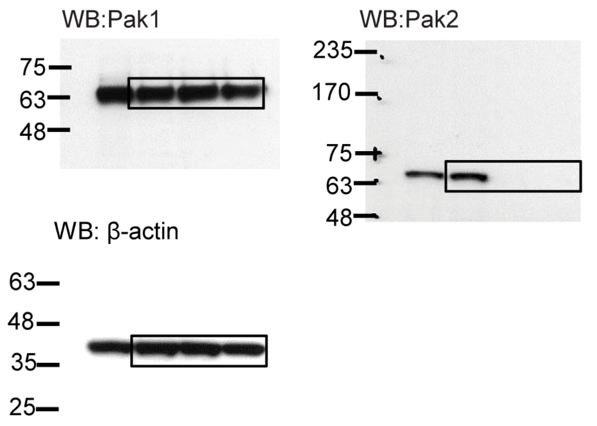

Supplement: Supplementary Information — Supplementary Figures 1-7. [file ncomms8437-s1.pdf]
